# A Psychometric Network Analysis of CHC Intelligence Measures: Implications for Research, Theory, and Interpretation of Broad CHC Scores “Beyond *g*”

**DOI:** 10.3390/jintelligence11010019

**Published:** 2023-01-16

**Authors:** Kevin S. McGrew, W. Joel Schneider, Scott L. Decker, Okan Bulut

**Affiliations:** 1 Institute for Applied Psychometrics, 1313 Pondview Lane E, St. Joseph, MN 56374, USA; 2 College of Education and Human Development, Temple University, Ritter Hall 358, Philadelphia, PA 19122, USA; 3 Applied Cognitive Neuropsychology Lab, Department of Psychology, University of South Carolina, Columbia, SC 29208, USA; 4 Centre for Research in Applied Measurement and Evaluation, University of Alberta, Edmonton, AB T6G 2G5, Canada

**Keywords:** CHC, Cattell–Horn–Cattell theory, intelligence, cognitive abilities, psychometric network analysis, process overlap theory, dynamic mutualism, factor analysis, general intelligence

## Abstract

For over a century, the structure of intelligence has been dominated by factor analytic methods that presume tests are indicators of latent entities (e.g., general intelligence or *g*). Recently, psychometric network methods and theories (e.g., process overlap theory; dynamic mutualism) have provided alternatives to *g*-centric factor models. However, few studies have investigated contemporary cognitive measures using network methods. We apply a Gaussian graphical network model to the age 9–19 standardization sample of the Woodcock–Johnson Tests of Cognitive Ability—Fourth Edition. Results support the primary broad abilities from the Cattell–Horn–Carroll (CHC) theory and suggest that the working memory–attentional control complex may be central to understanding a CHC network model of intelligence. Supplementary multidimensional scaling analyses indicate the existence of possible higher-order dimensions (PPIK; triadic theory; System I-II cognitive processing) as well as separate learning and retrieval aspects of long-term memory. Overall, the network approach offers a viable alternative to factor models with a *g*-centric bias (i.e., bifactor models) that have led to erroneous conclusions regarding the utility of broad CHC scores in test interpretation beyond the full-scale IQ, *g*.

## 1. Introduction

For the better part of the past three decades, there have been ongoing debates regarding the efficacy of cognitive ability testing within the field of school psychology (Farrell 2010; Jimerson et al. 2016; Kaufman et al. 2016). Researchers critical of cognitive ability testing have raised valid issues regarding the efficacy of clinical or actuarial interpretation (Canivez 2013b), the long-term stability of cognitive ability test profiles (Dombrowski et al. 2021; Farmer et al. 2021b), and the lack of empirically demonstrated cognitive ability test-based educational aptitude treatment interactions beyond psychometric *g* (Kranzler and Floyd 2020). These complex issues, as well as the debates surrounding the widespread pattern of strengths and weaknesses approach to specific learning disability (SLD) identification (Christo and Ponzuric 2017; Fiorello et al. 2014; Flanagan and Schneider 2016; Kranzler et al. 2016; Maki et al. 2022; McGill and Busse 2017; Taylor et al. 2017) are beyond the intended scope of this paper. Instead, this paper focuses on a central issue in the current cognitive ability test use debate—the relative importance of broad CHC Stratum II scores after the Stratum III general intelligence (*g*) construct variance is accounted for in cognitive–achievement relations research.

“The construct known as psychometric *g* is arguably the most important construct in all of psychology largely because of its ubiquitous presence in all tests of mental ability and its wide-ranging predictive validity for a great many socially significant variables” (Jensen 2002, p. 39). It is hard to ignore the robust replication of the existence of psychometric *g* as well as its documented dominance as the single best individual difference characteristic predictor of a wide variety of outcomes (Deary 2012; Lubinski 2004). The theoretical origin of the *g* factor derives from the consistent finding that cognitive ability tests covary positively (Protzko and Colom 2021a, 2021b). This empirical regularity is the *positive manifold* (Jensen 1998). The most dominant explanation of the positive manifold is that all cognitive ability tests correlate positively because they are all influenced by a general ability factor abbreviated as *g*. Spearman (1923, 1927) hypothesized that *g* is a general capacity to perceive relations among stimuli and deduce new information from those relations. Spearman speculated that individual differences in *g* derived from individual differences in a kind of “mental energy” derived from fundamental physiological properties of the brain (Hart and Spearman 1912).

Unfortunately, the theoretical meaning of *g*, as originally defined by Spearman (1927), is often misunderstood in intelligence testing structural research. This misunderstanding results in the frequent conflation of theoretical and psychometric *g*, a problem central to the debate regarding the importance of broad CHC composite scores in IQ testing.^1^

## 2. Literature Review

### 2.1. The beyond g Position: The Promise of Broad CHC Scores in Intelligence Testing Interpretation

The CHC theory (McGrew 1997, 2005, 2009) is widely accepted as an empirically defensible psychometric model of cognitive abilities (Alfonso et al. 2005; Schneider and McGrew 2012, 2018). This classic hierarchical cognitive ability model (Carroll 2003; Gustafsson 1984; Jensen 1998) places a psychometric *g* stratum III ability at the apex over multiple broad stratum II CHC abilities. Psychometric *g* is modeled as having both direct and indirect effects (mediated through broad CHC abilities) on achievement. In this paper this is referred to as the *mixed-g* model (*g* has a direct impact on broad CHC abilities [but no direct effect on the individual CHC tests], as well as *g* having possible indirect effects on achievement). An extensive number of mixed-*g* studies completed by Keith, Reynolds, and colleagues (e.g., Caemmerer et al. 2018, 2020; Floyd et al. 2003, 2007, 2008, 2009; Hajovsky et al. 2014; Keith 1999; Keith and Dunbar 1984; Keith and Reynolds 2010, 2018; McGrew et al. 1997; Meyer and Reynolds 2017; Niileksela et al. 2016; Reynolds et al. 2013; Reynolds and Keith 2017; Reynolds and Turek 2012; Taub et al. 2008; Vanderwood et al. 2002; hereafter called the Keith–Reynolds group) support a multi-factorial view of intelligence with a hierarchical psychometric *g* factor that does not focus on explaining as much common test variance as possible. While equivocal, Reynolds and Keith (2017) state that “the higher-order model is more in line with our current conception of human intelligence as it represents a system of interrelated latent constructs and not one in which *g* and [CHC] broad abilities operate entirely independently of one another” (p. 33).

The cognitive–achievement relations research completed by or modeled on Keith–Reynold’s hierarchical mixed-*g* approach, has reported that even when psychometric *g* is included in structural equation modeling (SEM) studies, aside from the strong direct effect of psychometric *g* on achievement, some broad CHC abilities demonstrate significant direct effects on specific achievement domains above and beyond the higher-order hierarchical psychometric *g* construct. Furthermore, psychometric *g* often demonstrates additional indirect effects mediated through broad CHC abilities. Multiple regression studies using the WJ-R and WJ III (Woodcock et al. 2001, 2007) standardization samples provide additional evidence supporting the developmental importance of specific CHC broad scores in the prediction of reading, writing, and math achievement in grade-school children (Evans et al. 2002; Floyd et al. 2003, 2008; McGrew and Hessler 1995; McGrew and Knopik 1993). These conclusions were replicated in the WJ IV norm data (Cormier et al. 2017a, 2017b; Cormier et al. 2016). Collectively these regression studies, which did not include a global IQ psychometric *g* proxy in the prediction models (i.e., no-*g* models), provided support for the measurement validity of specific CHC broad scores which have differential relationships with achievement domain scores (e.g., reading, writing, and math) mediated by development (McGrew and Wendling 2010). The mixed-*g* and no-*g* CHC model research supports the interpretation of broad CHC scores in educational settings.

### 2.2. The g-Centric Position: Broad CHC Scores Are of Trivial Value beyond the Global IQ Score

A strong pro-*g* IQ battery research program has recently achieved prominence in the field of cognitive assessment. A group of prolific scholars has repeatedly demonstrated that although the general factor of intelligence can be measured reliably in individuals and has predictive validity for significant outcomes, they believe they have shown that the smaller broad CHC scores cannot be measured reliably in individuals and provide only negligible amounts of helpful information (Beaujean et al. 2018; Canivez 2013a, 2013b; Canivez et al. 2016; Canivez and McGill 2016; Dombrowski et al. 2017; Dombrowski et al. 2019b; McGill and Busse 2017; McGill 2018; Nelson et al. 2013). This group’s research has resulted in calls for the de-implementation of many popular intelligence testing interpretation practices (Dombrowski et al. 2021; Farmer et al. 2021a, 2021b). In particular, the interpretation of broad CHC scores is considered a low-value practice. Hereafter we refer to this group collectively as the Canivez et al. group.

Compared to the hierarchical mixed-*g* (Keith–Reynolds group) research findings, conclusions based on the Canivez et al. pro-*g* bifactor research has, via their methods and procedures, pre-assumed that the statistically significant lion’s share of IQ battery test variance must be of the form of a dominant psychometric *g* factor (Decker et al. 2021). Psychometric *g* is modeled as having a direct effect on each test, unfettered by intermediate specific broad CHC abilities, and, when included in causal models, a powerful direct effect on achievement. Broad CHC scores are considered nothing more than a minor nuisance (i.e., left-over trivial residual sources of variance after psychometric *g* is accounted for) and should be avoided in interpretation (McGill et al. 2018). In this paper this is called the *g*-centric position regarding the intelligence testing interpretation research. How large this modest amount of additional explained variance must be before broad CHC scores are considered practically relevant and meaningful is an unresolved issue (McGill et al. 2018; Schneider et al. 2016); the “interpretation of these results is very much in the eye of the beholder” (McGill et al. 2018, p. 71).

The use of *g*-centric statistical models has not only conflated debates in theoretical research but has also extended to clinical application guidelines for using intelligence testing results on an individual basis. For example, Dombrowski et al. (2021) and Farmer et al. (2021b) appear to have discounted the plausibility of alternative views (i.e., broad CHC abilities with a higher-order psychometric *g* factor at the apex; the Keith–Reynolds group), in the promulgation of *g*-centric guidelines for clinical practice.

### 2.3. The Problem of Conflating Theoretical and Psychometric g

Contributing to the conflicting *g*-centric and mixed-*g* positions (regarding the interpretive value of broad CHC scores) is the largely unrecognized common practice of conflating theoretical and psychometric *g*. Psychometric *g* is the statistical extraction of a latent factor (via factor analysis) that accounts for the largest single source of common variance in a collection of cognitive abilities tests. It is an emergent property statistical index. Theoretical *g* refers to the underlying biological brain-based mechanism(s) that produce psychometric *g*. The global composite score from IQ test batteries is considered the best manifest proxy for psychometric *g*. The conflation of psychometric and theoretical *g* in IQ battery structural research ignores a simple fact—“general intelligence is not the primary *fact* of mainstream intelligence research; the primary fact is the *positive manifold….general intelligence is but one interpretation of that primary fact*” (Protzko and Colom 2021a, p. 2; emphasis added). As described later, contemporary intelligence and cognitive psychology research has provided reasonable and respected theories (e.g., dynamic mutualism; process overlap theory; wired cognition; attentional control), robust methods (psychometric network analysis), and supporting research (Burgoyne et al. 2022; Conway and Kovacs 2015; Kan et al. 2019; Kievit et al. 2016; Kovacs and Conway 2016, 2019; van der Maas et al. 2006, 2014, 2019) that accounts for the positive manifold of IQ test correlations in the absence of an underlying latent causal theoretical or psychometric *g* construct.

Fried (2020) and others (Eronen and Bringmann 2021; Kovacs and Conway 2019; Protzko and Colom 2021a, 2021b) have cogently explained why the cavalier use of *g*-like terms (e.g., *g* for general intelligence; *p* for general psychopathology) and the failure to differentiate between theoretical and psychometric models, contributes to the theory crises in psychology. This is particularly prevalent in psychological fields heavily dependent on structural analysis methods, such as IQ battery structural research. The divergent results of the Keith–Reynolds and Canivez et al. research groups, and the extensive replication of the *g*-centric findings of the Canivez et al. group in particular, are not likely to resolve the debate regarding the value of broad CHC cognitive scores in intelligence testing interpretations. Both groups seem entrenched in their respective factor analysis methods and assumptions. It is time intelligence testing structural research explores new and emerging data analysis methods and theories related to understanding the potential value of various scores from intelligence batteries.

### 2.4. The Application of Non-g Emergent Property Network Models to IQ Batteries

#### 2.4.1. Limitations of Common Cause Factor Models of Intelligence

Despite using different *g*-centric or mixed-*g* factor analysis methods, the Canivez et al. and Keith–Reynolds groups (and most intelligence structural researchers) have primarily used statistical methods from the same class of models for explaining the positive manifold of tests in IQ batteries—common cause models. A *common cause* model “embodies the idea that there is a common cause ‘out there’ that we ‘detect’ using factor analysis, and that should have an independently ascertainable identity in the form of, say, a variable defined on some biological substrate” (van der Maas et al. 2014, p. 13). Common cause IQ battery research almost always includes a psychometric *g* factor which is regularly conflated with theoretical *g*.

These classic common cause models have served admirably as the primary statistical descriptive and taxonomic-generating workhorses of twentieth-century intelligence research (Savi et al. 2021). They helped produce Carroll’s seminal treatise on the structure of human cognitive abilities, his three-stratum model of cognitive abilities, and the related CHC taxonomy of cognitive abilities (McGrew 2009). However, a major limitation of these methods is that regardless of how psychometric *g* is modeled (e.g., bifactor or hierarchical *g*), factor analysis methods prevent the exploration of causal mechanisms between cognitive constructs. Modeling *g* as the central causal entity “bars complex interactions both within the construct of intelligence itself and with its adjacent systems” (Savi et al. 2021, p. 5). This is clear when examining standard factor analysis path diagrams. Single-headed arrows lead directly from *g* (represented as a latent factor in the form of an oval) to the manifest test indicators (represented by rectangles) or to first-order broad cognitive ability factors (e.g., Gf, Gc, Gwm, etc.; latent factor ovals), which in turn have direct effect arrows from their respective cognitive abilities to the manifest test indicators. The origin and direction of the arrows leads to the inescapable conclusion that theoretical *g* is the primary causal mechanism that produces change in intellectual functioning. This explicitly implies that to improve intellectual functioning, theoretical *g* must be the primary target of intervention. However, attempts to improve theoretical *g* (whatever that may be) have largely been proven unsuccessful (when change is reflected in increases in the psychometric *g* variable, either represented by a global IQ score or a latent factor). These common cause *g*-dominated models also tend to promote (imply) the harmful and pessimistic notion of biological determinism and immutability of intellectual functioning (Holden and Hart 2021).

#### 2.4.2. The Potential of Psychometric Network Models of Intelligence Tests

Modern network-based models of intelligence (e.g., process overlap theory; dynamic mutualism) (Protzko and Colom 2021a, 2021b; van der Maas et al. 2019) eschew the assumption that the positive manifold among cognitive ability tests is due to latent unobserved common causes. Instead, network models (and psychometric network analysis methods, PNA) are based on the assumption that the positive definite nature of cognitive ability test correlations is the result of the non-linear interaction of multiple biological and psychological factors, sans the invocation of underlying latent common cause explanatory traits or factors (Hampshire et al. 2012; van der Maas et al. 2019). Much like the quantitative horsepower index of an automobile engine is an *emergent property* metric that reflects the by-product of the complex interaction of multiple engine systems (e.g., fuel, exhaust, ignition, combustion, cooling, lubrication, etc.), modern network cognitive ability theories postulate that psychometric *g* is the result of, and not the cause of, the positive manifold between IQ tests (Conway et al. 2021; Fried 2020; Hampshire et al. 2012; Kan et al. 2019; Kovacs and Conway 2016, 2019). Borsboom et al. (2021), Jones et al. (2018), Neal and Neal (2021), and Robinaugh et al. (2016) provide excellent overviews of PNA, from which we borrow extensively in our description below.

Briefly, in PNA, each psychological variable (i.e., tests and hypothesized abilities measured in our study) are represented by *nodes*. Weighted non-directional relations between nodes are identified and the strength of the test associations (*edges*; thicker edges represent stronger associations) is described with estimated statistical parameters (partial correlations coefficients). All patterns of pairwise conditional test relations are estimated as statistically independent of relations with all other tests.

**Figure 1:**
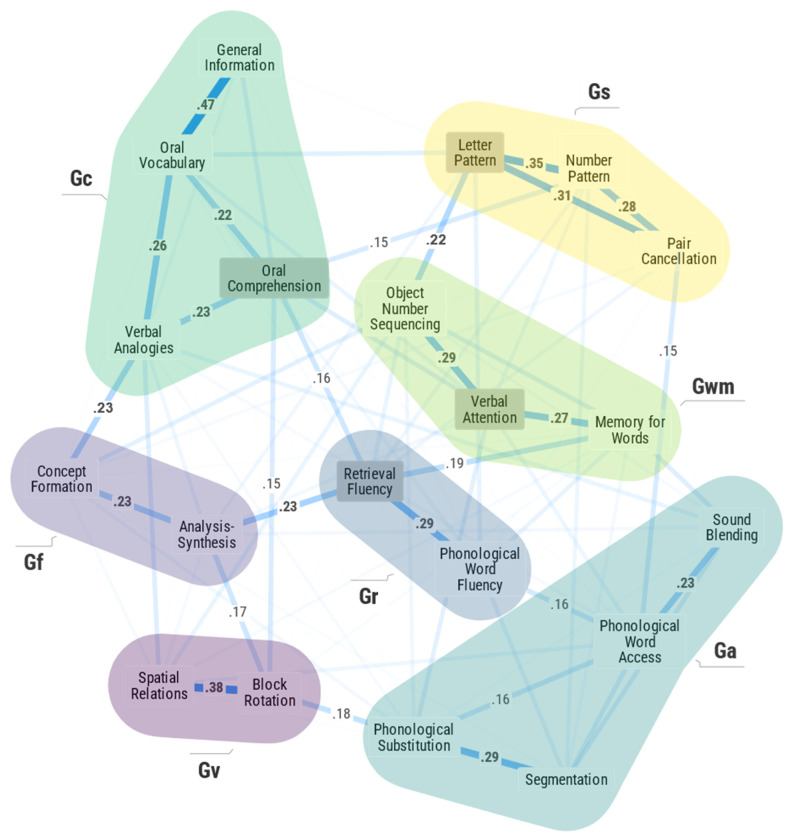
Weighted undirected network structure of 20 select WJ IV measures of seven broad CHC abilities in the primary network model. *Note.* Numbers are the edge weights (thickest lines) greater than or equal to .15. Edge weights greater than or equal to .20 in bold font. The four most central nodes are enclosed in gray boxes (see manuscript text).

The resulting IQ test network is characterized or described with tools from network science. PNA offers several benefits including: (1) the methods are atheoretical, data-driven, and exploratory in nature; (2) previously unknown insights regarding complex relations between interrelated variables can be analyzed simultaneously, including variables that can serve both as a predictor or predicted variable; (3) groupings or communities of test nodes akin to latent variable common cause factors can be identified; (4) the topography of the network is characterized by several network *centrality* metrics (closeness, betweenness, and strength, defined later in this paper) that reflect different relative importance characteristics of the tests in the global test network; (5) the methods provide parsimonious, reliable, and replicable results; (6) the methods provide strong exploratory descriptions of the relations between all subtests (all patterns of pairwise conditional test dependencies) in the absence of the statistical constraint to identify latent causal traits or factors; (7) powerful multidimensional visualization tools that display patterns of multivariate test dependencies and highlight key patterns of test associations (see Figure 1 as an example); (8) the models can be used to generate hypotheses regarding the causal mechanisms between abilities measured by tests, thus offering potential insights for targets of intervention (e.g., control or driver nodes) within the network; and (9) “because network models for multivariate data explicitly represent pairwise interactions between components in a system, they form a natural bridge from data analytics to theory formation based on network science principles” (Borsboom et al. 2021, p. 15). More specific details regarding PNA methods and interpretations are presented in the materials and methods section in this paper.

PNA methods are, at face value, exploratory and descriptive—they do not directly suggest causal mechanisms in the psychological network. However, this is an upside of PNA models *when combined with substantive knowledge and network science tools*. The primary value of these descriptive models is their ability to function as a bridge to theory formation and the ability to hypothesize, and empirically test or statistically simulate, potential causal mechanisms in the network (Borsboom et al. 2021; Haslbeck et al. 2021). In contrast, classic statistical prediction models such as multiple regression provide few hints regarding possible complex causal relations between and among variables in a regression model. SEM causal models have the potential to illuminate causal relations between and among broad CHC abilities. However, currently no comprehensive CHC explanatory causal SEM-based models have been validated. Although PNA models are primarily descriptive, they can be investigated with various tools from network science (e.g., exploratory and confirmatory PNA; exploratory stepwise search algorithms to guide the removal or addition of nodes to improve the model; *in silico* mathematical simulations where changes in network nodes are statistically modified [or constrained] to see how the effect propagates through the entire network—and thus, potentially identify causal mechanisms in the network; etc.) (Epskamp et al. 2017; Haslbeck et al. 2021; Lunansky et al. 2022).

PNA models of cognitive abilities can assume a pivotal role in improving CHC cognitive–achievement relations SEM modeling research as it can act “as a natural interface between correlation and causality…. [as] the typical attempt to determine directed SEMs from correlation structures in fact appears somewhat haphazard in psychology, a historical accident in a field that has been prematurely directed to hypothesis testing at the expense of systematic exploration (Epskamp et al. 2017, pp. 924–25). PNA methods can facilitate CHC SEM modeling via the systematic identification of relations between multiple variables unfettered by concerns for direct causal relations or reciprocal causation between and among latent common causes (Epskamp et al. 2017). PNA of CHC tests or factor analyses derived broad CHC scores can serve as a new lens by which to identify potential key levers for understanding relations between cognitive abilities, intellectual functioning, and school achievement.

In summary, no-*g* PNA methods differ from the common cause *g*-centric and mixed-*g* factor analysis as they can provide new insights regarding the relations between and among tests of broad CHC cognitive and achievement abilities that may help resolve the current debate regarding the utility of broad CHC scores. This promise is captured by Savi et al. (2021) who have stated that modern network theories and methods “will dominate the twenty first [century of human intelligence research]” (Savi et al. 2021; p. 1).

#### 2.4.3. Prior PNA of IQ Batteries

To date, only a few studies have analyzed contemporary IQ batteries with PNA. These include PNA of the Hungarian and US WAIS-IV (Schmank et al. 2019, 2021), US WAIS-III (van der Maas et al. 2017), and the WJ IV (Bulut et al. 2021). These PNA IQ battery studies have been confined to the universe (or subset) of measures specific to each IQ battery. None have been grounded in the a priori selection of measures as per contemporary CHC or other intelligence theories. These studies have demonstrated that PNA of IQ batteries can produce network results consistent with no-*g* theoretical models of intelligence (e.g., dynamic mutualism and process overlap) and, when compared with latent variable factor models, the network models identify equally plausible representations of the multidimensional structure of IQ tests (Bulut et al. 2021). The PNA models typically identify the same groupings or communities of tests akin to the latent variable common cause factor analysis-based broad CHC score indices (e.g., crystallized intelligence, fluid reasoning, working memory, processing speed in the WAIS-IV; Schmank et al. 2021). Absent from this small collection of IQ battery PNA studies are attempts to link the resulting network model and test network centrality metrics to intelligence testing interpretation. Although certain tests have been identified as central, gatekeeper, or bridge measures in an IQ battery network (Bulut et al. 2021), how these most central tests (and abilities they measure) might operate as hypothesized causal mechanism target systems (Haslbeck et al. 2021) to inform theory building and possible interventions, has been absent.

Although not focused on any specific IQ battery or comprehensive intelligence theory, studies with select sets of cognitive and or achievement tests have demonstrated how PNA descriptive models can provide support for potential causal mechanisms or theories of cognitive deficits in first episode psychosis (Sánchez-Torres et al. 2022), the Simple View of Reading model (SVR; Angelelli et al. 2021), a multi-level model of learning skills (Zoccolotti et al. 2021), and the age-differentiated relations between cognitive efficiency abilities (i.e., inhibition, working memory, fluid intelligence, and speeded attention) (Neubeck et al. 2022).

### 2.5. Current Study

The goal of the current research is to move beyond the common cause factor analysis-based *g*-debates in IQ battery structural research to explore modern no-*g* complementary methods (PNA) for analyzing IQ batteries. The purposes of the current study are fourfold. First, the construct validity of the CHC model of cognitive abilities is evaluated with no-*g* PNA methods. The benefits (and cautions and limitations) of applying PNA methods to the structural analysis of IQ tests are demonstrated vis-à-vis the exploration of a carefully selected set of measures representing the broad abilities of the CHC cognitive ability theory in a large, nationally representative sample of school-aged children. Second, the ability to minimize the “boundary specification problem” (i.e., the impact of incomplete or unmeasured nodes or measures on the robustness of the final PNA model; Neal and Neal 2021) in psychological networks is demonstrated by: (1) using the consensus model of cognitive abilities (CHC) for the a priori selection of ability measures for the primary CHC analysis and (2) supplementing the primary network analyses with three secondary sensitivity model analyses. Third, the potential usefulness of PNA centrality metrics for applied IQ battery interpretations is explored. Finally, complimentary network visualization methods (e.g., multidimensional scaling and minimal spanning trees) are presented to minimize the potential pitfalls in the interpretation of the most central psychometric network topological features (Jones et al. 2018; Letina et al. 2019).

## 3. Materials and Methods

### 3.1. Participants

The sample consisted of a school-aged (ages 9–19) subsample (*n* = 3258) of the US nationally representative norming study of the WJ IV, a battery of tests comprised of four components—cognitive (COG), oral language (OL), achievement (ACH), and Early Cognitive and Academic Development (ECAD) (McGrew et al. 2014; Schrank et al. 2015). The WJ IV is an individually administered comprehensive system of tests designed to measure various cognitive, oral language, and achievement abilities. The WJ IV battery is aligned with CHC theory and displays strong psychometric qualities (McGrew et al. 2014; Reynolds and Niileksela 2015). The age 9–19 subsample was selected since it covered the same age ranges independently analyzed with more traditional common cause exploratory and confirmatory factor analysis models (both bifactor and hierarchical *g*) of different subsets of the complete WJ IV battery (Dombrowski et al. 2018a, 2018b; Niileksela et al. 2016). See Dombrowski et al. (2019a) and Villarreal (2015) for additional independent reviews of the psychometric characteristics of the WJ IV.

The complete WJ IV standardization sample is representative of the US population (as per the 2010 US Census) in terms of individual (e.g., sex, race, and parent education) and community variables (e.g., census region and community type). The age 9–19 school-age sample had a mean age of 14.3 years (*SD* = 3.2). The gender × race composition of the sample, using the sampling variables reported and defined in the WJ IV technical manual (McGrew et al. 2014), is summarized in Table 1. Additional detailed demographic information for the school-age samples is available in the WJ IV technical manual.

### 3.2. Measures

Since network topology metrics can change significantly if certain measure nodes are missing (i.e., are unmeasured) from the relevant universe of psychological constructs (Neal and Neal 2021), a careful measure selection process was used to operationalize each broad CHC ability with two to four of the most valid WJ IV measures for each broad CHC broad ability.^2^ This process ensured that a minimally complete set of measure nodes relevant to understanding the CHC model are included, and measure nodes superfluous to understanding CHC constructs are excluded (Bringmann et al. 2019, 2022). Minimizing the boundary specification problem, while concurrently increasing the generalizability of the PNA results, was achieved through the systematic and careful measure selection process described in Supplementary Materials Section S1.

The final set of 20 broad CHC measures used in the primary analysis, as classified by Schneider (2016), Schneider and McGrew (2018), and our own CHC analysis, were: Fluid Reasoning-Gf (Concept Formation, CONFRM; Analysis-Synthesis, ANLSYN), Comprehension–Knowledge-Gc (Oral Vocabulary, ORLVOC, General Information, GENINF; Oral Comprehension, ORLCMP; Verbal Analogies, VRBANL), Visual Processing-Gv (Spatial Relations, VZSPRL; Block Rotation, VZBLKR), Auditory Processing-Ga (Segmentation, SEGMNT; Sound Blending, SNDBLN; Phonological Processing-Word Access, PPACC; Phonological Processing-Word Substitution, PPSUB), Short-term Working Memory-Gwm (Verbal Attention, VRBATN; Objective-Number Sequencing, OBJNUM; Memory for Words, MEMWRD), Retrieval Fluency-Gr (Retrieval Fluency, RETFLU; Phonological Processing–Word Fluency, PPLU), Processing Speed-Gs (Letter–Pattern Matching, LETPAT; Number Pattern Matching, NUMPAT; Pair Cancellation; PAIRCN). It is important to note that this set of WJ IV measures differs from the Bulut et al. (2021) PNA study that was restricted to the primary 14 COG measures in the WJ IV. The current study selected the purest CHC measures from across the WJ IV COG, OL, and ECAD components.

As explained in Supplementary Materials Section S1 three of the primary WJ IV COG measures (Number Series, Story Recall, Visual–Auditory Learning) were excluded from the 20-measure primary model as the extant research has suggested that they are either factorially complex indicators of CHC constructs (e.g., Number Series, Gf and Gq) or post-publication reviews of the WJ IV questioned whether they were strong indicators of specific broad CHC abilities (e.g., Visual–Auditory Learning may be a poor indicator of Gl; see Schneider 2016). A 23-measure sensitivity model added the Number Series (NUMSER) back into the analysis together with two measures of Gq (Calculation, CALC; Applied Problems, APPROB). An additional 25-measure sensitivity analysis model included the WJ IV Gl measures that had been excluded from the 20- and 23-measure analyses (Visual–Auditory Learning, VAL; Story Recall, STYREC). Detailed descriptions of the measures and associated psychometric information can be found in McGrew et al. (2014) and Schrank et al. (2015).

### 3.3. Data Analysis

#### 3.3.1. Score Metric and Analysis Software

The publisher-provided age-based standard scores for each measure would have been the ideal metric for analysis. However, the WJ IV does not provide standard scores for subtest measures. In their absence, the WJ IV *W*-score, which is based on a direct transformation of the Rasch logit scale (with a center of 500 points at the age of 10 years), was the selected metric (McGrew et al. 2014). To allow for the analysis of the measure scores across a wide developmental age range, residual *W*-scores were created by statistically removing chronological age variance (in months; CAMOS) from the scores (using CAMOS and CAMOS^2^ terms). The resulting raw data file of residual *W*-scores was used for the analysis. The statistical analysis was completed with the open-source *JASP* (v.0.16.3; JASP Team 2022) network analysis software program module supported by the University of Amsterdam (https://jasp-stats.org/; accessed on 4 October 2021). In addition, non-network measure information was calculated to investigate the relations between PNA centrality metrics and more commonly reported psychometric information for cognitive measures (i.e., psychometric *g*-loadings). Individual measure psychometric *g*-loadings were calculated for the 20-measure primary model by calculating the first unrotated component or factor in principal component and principle-axes factor analysis of the zero-order correlations.

#### 3.3.2. PNA Methods

The *JASP* network module generated the CHC PNA Gaussian graphical models with the *EBICglasso* estimator (Epskamp and Fried 2018; Friedman et al. 2008). The *JASP* network module default parameters were used for model estimation. The *LASSO regularization* technique was used to estimate the edge weights (partial correlations), a statistical method that emphasizes model parsimony over complexity (Epskamp and Fried 2018). This technique invokes a penalty for extremely dense and complex networks. The LASSO technique removes non-significant edges by estimating them to be zero. Thus, the final network is sparser and only includes what are called *non-zero* node (test) edges. The *JASP* network module provided the network metrics of betweenness, closeness, and strength that collectively described different centrality characteristics of the network typology (Borsboom et al. 2021; Bulut et al. 2021; Jones et al. 2018; Neal and Neal 2021; Robinaugh et al. 2016).

The closeness index represents how close a measure node is, on average, to all other measure nodes. Closeness quantifies the distance relationship of a specific measure node to all other measure nodes by computing the average of the shortest path lengths to all other measure nodes. High closeness suggests that a measure node can “communicate” in an optimal or efficient manner with other measure nodes (Bringmann et al. 2019). The betweenness index quantifies how frequently a measure node lies on the shortest path connecting any two other nodes and often suggests which measure nodes function as “middlemen” or “gatekeepers” between non-adjacent measure nodes (Bringmann et al. 2019; Bulut et al. 2021; Jones et al. 2018).

The *strength* index conveys how strongly, on average, a specific measure node is connected or conditionally associated with all other measure nodes in the network. Strength is an overall measure of connectedness based on the sum of the absolute values of all edges connected to a measure node. In general, the betweenness and closeness indices have been most useful in social network analysis, while the strength index has been emphasized in psychopathology networks (Jones et al. 2018). Recent large-scale network simulations have suggested that the strength centrality metric demonstrates the highest correlation between true and simulated networks, followed next by measures of closeness and then betweenness indices (Isvoranu and Epskamp 2021). The paucity of network research with IQ tests suggests a cautious interpretation of the different network centrality metrics and a need to identify which indices may be most relevant to the network analysis of IQ battery data.

Network figures must be interpreted with great care, as by presenting multidimensional data in two-dimensional space, “visual interpretation of the position of nodes can easily lead one astray” (Jones et al. 2018, p. 2). Jones et al. (2018) noted three potential “visual (mis)interpretations of networks” based on false assumptions regarding: (1) node association strength, (2) the relative placement of nodes on the networks X and Y axis, and (3) node centrality not being represented in the middle of the visual network. Several complimentary low-dimensional visualization methods and supplementary sensitivity model analysis of the same dataset have been recommended (Jones et al. 2018; Letina et al. 2019).

Of the recommended complimentary methods, multidimensional scaling (MDS) and the minimal spanning tree (MST) algorithm were selected to help identify the key “skeleton” or “backbone” structure of the network (Letina et al. 2019). The MST algorithm uses a minimal number of edges or links to connect the measure nodes in a visual–graphic network. The complimentary MDS and MST analyses were combined in single 2-D MDS models (Guttman Radex Model; Cohen et al. 2006; Guttman 1954; Guttman and Greenbaum 1998). The MDS and MST methods portray the distances (degree of association) between network measure nodes more accurately than visually complex PNA model figures (Jones et al. 2018; Letina et al. 2019). The MDS and MST data analysis procedures were completed with the *SYSTAT* v13.1 statistical software (Wilkinson 2010).

To ascertain the impact of the a priori exclusion of the Number Series, a 23-measure secondary sensitivity network analysis was completed that included Number Series together with two WJ IV math achievement (Gq) measures. This included repeating the PNA analysis and the generation of the network figures. The primary and secondary sensitivity network analysis model centrality metrics, and associated network figures, were inspected to determine if the empirical network characteristics of any measures changed, and if they did, to identify those measures whose network metrics were most robust across the 20-measure primary and 23-measure sensitivity analysis. Measure node information external to the PNA network was examined by correlating traditional psychometric *g*-loadings for the primary 20 CHC measures with the network centrality metric statistics from the 20-measure model (see Supplementary Materials Section S6). Finally, a second 25-measure sensitivity analysis model was completed as a post hoc verification of the a priori decision to exclude the WJ IV Gl measures (Visual-Auditory Learning and Story Recall) in the primary model. Only the network and MDS + MST network figures are presented for the 25-measure secondary sensitivity analyses.

## 4. Results

### 4.1. PNA Models

**Figure 2:**
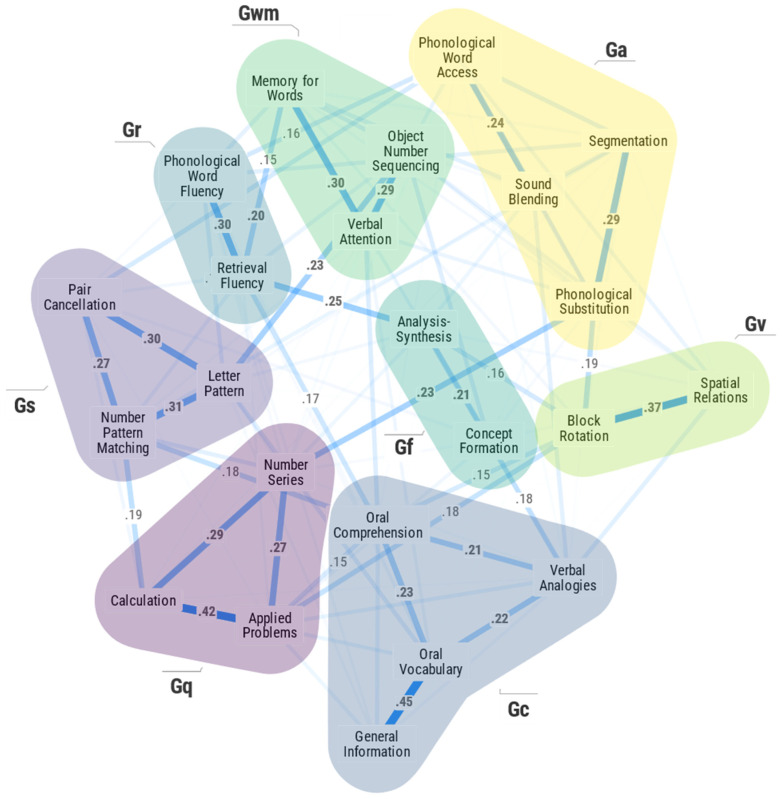
Weighted undirected network structure of 23 select WJ IV measures of eight broad CHC abilities in the secondary sensitivity network model.

The 20-measure primary network model is presented in Figure 1. Figure 1, as well as Figure 2 in this text, were crafted to be more presentation quality figures (when compared to that typically provided by most PNA software programs) and to include select information (*JASP* network centrality metrics and weights matrix table information) to aid interpretation. In Figure 1, the color of each measure node indicates the broad CHC ability defined by the respective WJ IV measures. The links (edges) represent the significant partial correlation weights between measure nodes. In the primary 20-measure model, 149 of 190 possible network edges were identified as non-zero edges (sparsity index = .22). The relatively large number of network edges is likely due to the high power of the statistical analyses due to the large sample size (*n* = 3258). Many of the edge weights were close to zero and thus not suggestive of strong network associations warranting interpretation. This can be seen in a quantile distribution plot of the network edge weights (Supplementary Materials Section S3) where approximately 25% of the edge weights were zero and approximately 50% of the edge weights were equal to or less than 0.05. As seen in Figure 1, only 17 edge weights (8.9%) were .20 or greater, and 9 more edges (4.7%) ranged from .15 to .19. At first, edge weights of .10 or greater were included in the PNA figures for interpretation. However, for the model in Figure 1, this nearly doubled the number of paths for interpretation from 26 to 51. This less stringent edge weight criterion produced an extremely complex figure that was difficult to interpret. The final criterion (i.e., edge weights greater than or equal to .15) produced a more conservative, parsimonious, and sparse model which was judged to be more appropriate as a first attempt to explore a CHC PNA model of intelligence (see Costantini et al. 2015 for discussion of the tradeoffs between strict adherence to strict statistical rules and the need for parsimony and sparser networks that are more theoretically interpretable).

Each edge weight can be interpreted based on its sign (positive or negative) and magnitude. An edge weight represents the degree of association left between two tests after conditioning on all other variables (i.e., removing all variance associated with other tests in the network). For example, the edge weights of .47 between Oral Vocabulary and General Information and .26 between Oral Vocabulary and Verbal Analogies are both positive, indicating a positive interaction between the nodes independent of relations with the 18 other measures in the network. However, Oral Vocabulary indicates a stronger relationship with General Information (.47) than it does with Verbal Analogies (.26). This suggests that the nodes of Oral Vocabulary and General Information influence each other more easily than the nodes of Oral Vocabulary and Verbal Analogies. In general, edge weights within traits are stronger than edge weights between other traits (Costantini et al. 2015). The primary network model (in Figure 1) was characterized as having stable centrality metrics as determined by *case-dropping subset bootstrap* methods (Epskamp et al. 2018a; see case-dropping subset bootstrap centrality metric analysis and discussion in Supplementary Materials Section S4).

The current results support at least seven broad CHC abilities or network communities (Gf, Gc, Gv, Ga, Gwm, Gr, and Gs). The evidence is robust as PNA methods do not specify an a priori model to be established like confirmatory factor analysis. Moreover, in PNA, the identified network structure is freed from the powerful a priori statistical constraint of specifying a dominant psychometric *g* factor as reflected in *g*-centric models of factor analysis. The confirmation of the broad Gf, Gc, Gv, Ga, Gwm, Gr, and Gs abilities in the current PNA analysis of the WJ IV measures is at variance from Dombrowski et al.’s (Dombrowski et al. 2018a, 2018b) failure to identify all of these broad CHC abilities in their analyses of similar school-age samples of the WJ IV norm data (although not using the exact same measures as in the current study). The difference in findings and conclusions is most likely the result of the current study method eschewing the inclusion of a psychometric *g* construct, while the Dombrowski et al. (Dombrowski et al. 2018a, 2018b) studies use methods where the psychometric *g* factor is focal to their analysis and interpretation of the results.

The “new” Gr broad ability in contemporary CHC theory (Schneider and McGrew 2018) is not that new—it was featured in two of CHC’s source theories (Carroll 1993; Cattell 1971, 1987). The Gr construct was supported here by the strong association between Retrieval Fluency and Phonological Processing: Word Fluency. The thickness of the links (weights) in the Gs (Letter–Pattern Matching, Number–Pattern Matching, and Pair Cancellation) and Gv (Spatial Relations and Block Rotation) sets of measures suggest these measures form tight and cohesive broad Gs and Gv dimensions. The Gc (Oral Vocabulary, General Information, Oral Comprehension, Verbal Analogies), Gf (Analysis–Synthesis and Concept Formation), and Gwm (Object–Number Sequencing, Verbal Attention, Memory for Words) communities also demonstrated consistent and strong within-domain associations, supporting the validity of these broad CHC broad abilities. Within Gc, Oral Vocabulary and General Information had a relatively stronger association (.47) than with the other within Gc measures (.22 to .26). This finding supports Schneider’s (2016) suggestion that the WJ IV General Information test “is more of a vocabulary test than a general knowledge test” (pp. 193–94). Although the Ga dimension is also distinct in the global network, the strength of pairs of relations (Phonological Processing–Word Substitution and Segmentation—.29; Sound Blending and Phonological Processing–Word Access—.23) suggest this dimension may be less cohesive or might have a substructure warranting further study.

The 23-measure secondary sensitivity analysis model (including Number Series and two Gq achievement tests) is presented in Figure 2. At a global topological level, the model retains the broad CHC abilities from the primary model (Gc, Gf, Gv, Ga, Gwm, Gr, and Gs) and integrates a tight and cohesive broad Gq ability dimension (Number Series, Calculation and Applied Problems). This provides evidence for the robustness of the primary 20-measure seven broad CHC ability model. Consistent with Schneider’s (2016) analysis, the Number Series measure is more strongly associated with the other Gq tests (.27 with Applied Problems; .29 with Calculation) than it is with the Gf tests (.06 with Concept Formation; .09 with Analysis–Synthesis). This supports the 20-measure primary CHC models a priori theory-based exclusion of the Number Series measure.

The spatial configuration of network nodes should not be overinterpreted, but it is intriguing that the node layout in Figure 1 and Figure 2 is consistent with Cattell’s (1971, 1987, 1998) triadic theory in that the higher-order groupings of general capacities (Gf, Gwm, Gs, and Gr), provincial powers (Gv and Ga), and agencies (Gc and Gq) are distinguishable.

### 4.2. PNA Model Centrality Metrics

The PNA centrality metrics for the primary 20- and 23-measure secondary sensitivity analysis network models are presented in Table 2. PNA centrality metrics are frequently presented in the form of standardized *z*-scores in graphs (e.g., see Bulut et al. 2021). Although visually informative, we instead present the relative centrality values that rescale each centrality index, so the strongest measure node has a value of 1.0 (intelligence testing researchers are more familiar with scaled psychometric information, e.g., *g*-loadings, factor loadings, reliabilities). The rescaling to the relative metric does not impact interpretation as reflected in a unity (1.0) correlation between the respective standardized *z* and relative centrality metrics. The measures are also organized by broad CHC abilities to facilitate the interpretation of potentially important domain-specific findings.

Across both the primary and secondary sensitivity analysis and all three-centrality metrics, the Oral Comprehension (Gc) measure was the most central measure in the two CHC networks. It is followed next by the Letter–Pattern Matching (Gs) measure, which was also identified as a central measure by Bulut et al. (2021). Of interest was the finding that in the 23-measure model, although Number Series was a top four central node (.96) as per the strength metric, across all three centrality metrics Number Series (the most consistently central node in Bulut et al. 2021) was replaced by Oral Comprehension as the most central measure. In the Bulut et al. (2021) study, the Gc measure of Oral Vocabulary was also one of the top three central network measures, suggesting that the prominence of Oral Comprehension in the current analysis may simply reflect the importance of the entire broad Gc ability domain. These findings reinforce the need to carefully represent all relevant measures that are practically feasible in a *theory-based* cognitive network to minimize results that may reflect boundary specification issues. In the primary network analysis model, the next most central measures, as per the closeness metric and particularly the strength metric, were Verbal Attention (Gwm) and Retrieval Fluency (Gr).

**Figure 3:**
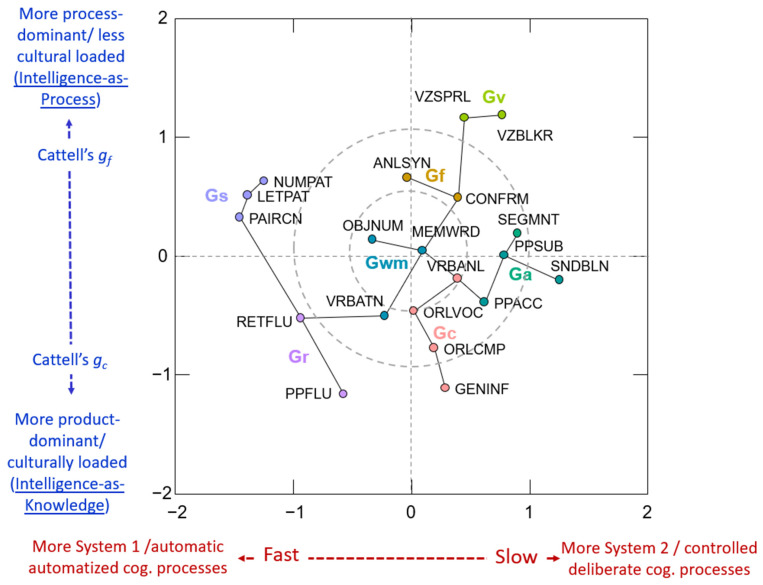
2-D MDS analysis (Guttman Radex) of a 20-measure primary model connected by the MST algorithm.

**Figure 4:**
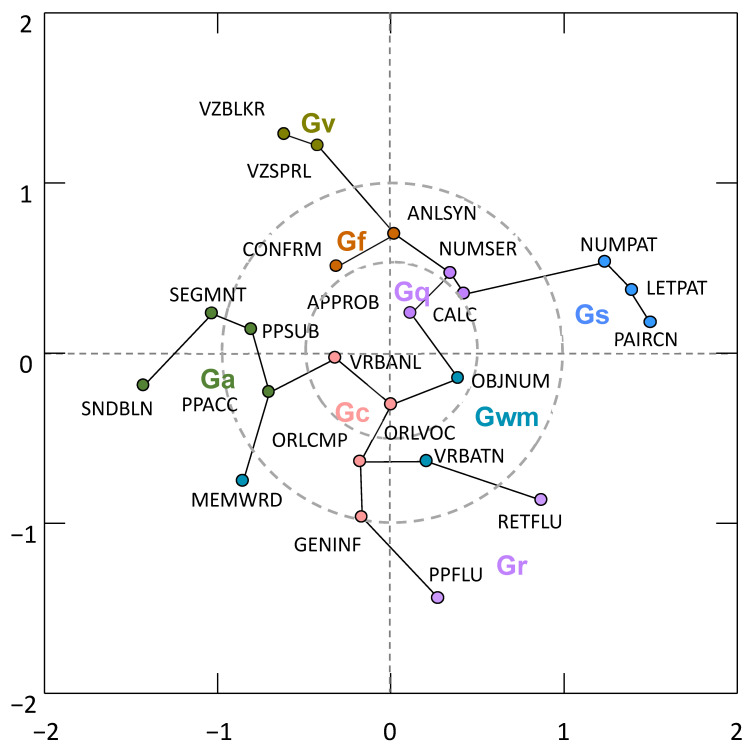
2-D MDS analysis (Guttman Radex) of a 23-measure secondary sensitivity model connected by the MST algorithm.

Table 3 summarizes correlations between the three network centrality metrics for the 20 primary measures calculated in both the primary and secondary sensitivity analysis models. The three bolded values (betweenness *r* = .84; closeness *r* = .88; strength *r* = .93) indicate that by including Number Series and Gq measures, the relative centrality of each measure did not change dramatically in the sensitivity analysis. This provides general evidence for the robustness of the seven broad CHC ability models. This is particularly true for the strength indices across both models (*r* = .93), suggesting that the strength centrality metric may be most robust to boundary specification issues. The strong, albeit slightly lower, values for betweenness (.84) and closeness (.88) suggest these network metrics may be slightly more sensitive to the inclusion or exclusion of other measures in a model under investigation. Of particular interest is the observation in Table 2 that although correlated at .84, and thus suggesting a consistent relative ordering of betweenness indices for the 20 primary measures, the absolute magnitude of the betweenness indices of the measured variables dropped dramatically in the 23-measure sensitivity analysis. This indicates that by introducing Number Series and the broad Gq ability in the analysis, all CHC measures became more distant from one another—that is, they were “pushed farther apart” in the network that included Gq measures. This suggests the hypothesis that when analyzing IQ measures with PNA, the inclusion of more traditional academic acquired knowledge measures (i.e., school achievement) may diminish the overall strength of association among and between cognitive measures. This is not immediately apparent from a review of the global networks in Figure 1 and Figure 3. This is clearer in the supplementary MDS + MST network Figure 3 and Figure 4.

Finally, the low to moderate correlations (*r* = −.24 to .43) between the corresponding centrality metrics (from both models) with traditional psychometric *g*-loadings indicates that PNA metrics provide valuable information regarding the relations of intelligence measures not captured by a psychometric *g*-factor. This finding is consistent with the fact that PNA is conceptually akin to common cause bifactor models that remove the large psychometric *g* variance before exploring secondary residual variance-based common factors (Protzko and Colom 2021b). As per Jensen’s (1998) principle of adequate psychometric sampling, the linear combination of the 18 remaining diverse measures serves as a proxy of psychometric *g*. To verify this assumption, seven different proxies of psychometric *g* were calculated for each of the seven broad CHC abilities. This represents a subset of the much larger number of total possible psychometric *g* proxies for all pairs of 20 measures. For example, for Gf, a non-Gf proxy of psychometric *g* was the arithmetic average of the 18 non-Gf (Analysis–Synthesis, Concept Formation) measures. Seven such composite scores were calculated. The correlations between these seven composite measures ranged from .93 to .99. One can therefore assume that when conducting PNA with IQ tests, the resulting network represents the relations between measures purged of shared or common variance. This is not to be confused with the assumption that psychometric *g* is essential to PNA research. As described previously in this paper, PNA models of intelligence measures eschew the concept of an underlying latent trait or cause (psychometric or theoretical *g*).

### 4.3. Complimentary MDS and MST Analysis

Figure 3 and Figure 4 present the 2D MDS models for the 20- and 23-measure primary and secondary sensitivity analysis models where the measure nodes are connected by the MST algorithm. In these figures, each WJ IV measure is presented in a Cartesian 2D coordinate system. The proximity of measures to each other indicates how similar the measures are. By comparing Figure 1 and Figure 4, four primary conclusions are noted. First, the presence of the seven broad CHC abilities is validated. The careful a priori selection of measures as per CHC theory resulted in seven distinct communities of broad CHC abilities. The within-broad CHC ability contiguous connectedness of measure nodes in Figure 4, together with the distinct presence of the broad Gc, Gf, Gv, Ga, Gwm, Gr, and Gs ability domains, provides PNA support for CHC theory. Second, several frequently misinterpreted features of PNA model figures identified by Jones et al. (2018) were present in the results. In Figure 1, the Gs measures appear closer to the Gc and Gwm measures. However, in Figure 2, it is obvious that the Gs measures are close to the Gwm and Gr measures, not the Gwm and Gc measures. Third, the two most central measure nodes (Oral Comprehension, Letter-Pattern Matching) are not located topographically as the most central measure nodes in Figure 1. In fact, Letter-Pattern Matching is visually located on the outward periphery of Figure 1. This serves to remind researchers that network centrality indices reflect the centrality of measures as a function of the quantity and strength of measure node links to the central measure nodes, not their geographic location in the global network figure.

Fourth, attempting to visually interpret the global multidimensional network model in Figure 1 (in this case seven CHC dimensions) in 2-D space can be a fool’s errand. The complimentary 2-D MDS + MST configuration in Figure 3 revealed an unexpected and potentially important insight regarding a higher-order (or more fundamental or skeletal) organization of the intelligence measure network in the model in Figure 1. This hypothesized interpretation is presented in the labeling of the two MDS dimensions (X and Y axes) in Figure 3. Briefly, the WJ IV measures are ordered from left-to-right (*X*-axis) as per an emphasis on System 1, automatic, or more automatized cognitive functions to more System 2, controlled, or more deliberate cognitive functions (Barrouillet 2011; De Neys and Pennycook 2019; Kahneman 2011). The second dimension (*Y*-axis) orders WJ IV measures from top-to-bottom as measures that resemble Cattell’s *g_f_* or Ackerman’s *intelligence-as-process* to Cattell’s *g_c_* or Ackerman’s (1996, 2018) *intelligence-as-knowledge* (particularly auditory–linguistic based knowledge).^3^ This insight into possible new lenses from which to interpret intelligence measures and dimensions within the CHC framework is not apparent from the fully dimensional global network model in Figure 1. The rationale for these interpretations is presented in the discussion section of this article.

Finally, Figure 4, when compared to Figure 3, suggests that the deliberate a priori theoretical and research-based selection of construct indicators may be a promising strategy to minimize the boundary specification problem in psychological network models. Although the proximity of Number Series and Gq math measures (Calculation and Applied Problems) to the Analysis–Synthesis measure (a miniature-controlled learning math logic test) in Figure 2 supports the use of Number Series as an indicator of Gf (RQ or quantitative reasoning) in the WJ IV, it may come at a cost to the integrity of the total CHC network model. The within-CHC broad abilities contiguously connected measure configurations reflected in Figure 2 are “broken” or “disrupted “in Figure 3 where the three Gwm measures (Object–Number Sequencing, Verbal Attention, Memory for Words) are no longer connected contiguously while disrupting the previously clearly connected within-CHC broad Gr and Ga community dimension measures. The Memory for Word measure is placed much farther away from the other Gwm measures in the network model in Figure 4. An additional secondary sensitivity network analysis model, including the a priori excluded WJ IV Gl measures, also supported the exclusion of these measures as the primary model was similarly degraded by their inclusion. The network and MDS + MST figures for this 25-measure supplementary sensitivity analysis are presented in the Supplementary Materials Section S5.

## 5. Discussion

The results of this study have implications for the following: (1) theories of intelligence and cognitive abilities, (2) potential CHC network-based intervention research, (3) the use and interpretation of broad CHC scores in intelligence testing in general and select WJ IV tests, and (4) methodological issues when investigating and reporting PNA results for tests in intelligence batteries.

### 5.1. Implications for Theories of Intelligence and Cognitive Abilities

#### 5.1.1. Implications for CHC Theory

The validity of the CHC theory and IQ battery test measures of CHC broad cognitive abilities has been questioned by research largely completed by the Canivez et al. *g*-centric research group. The current study is consistent with the Keith–Reynolds (and other researchers, e.g., see Schneider and McGrew 2012, 2018) mixed-*g* research that supports a multidimensional CHC model of intelligence with broad CHC abilities of Gc, Gf, Gv, Ga, Gwm, Gr, Gs, and Gq—sans the need to include to a unitary latent psychometric general intelligence (*g*) construct to explain the positive manifold of IQ test correlations. This finding is also consistent with Horn’s no-*g* position and emergent property model theories of intelligence (process overlap theory; dynamic mutualism; wired intelligence).

The recent recommendation to cleave Gr (retrieval fluency) from Glr (long-term retrieval; Schneider and McGrew 2018) was supported. The current results raise questions regarding the measurement and validity of Gl (learning efficiency) as operationalized by the two WJ IV Gl tests (Visual-Auditory Learning; Story Recall). The construct validity of the broad Gl ability itself requires further investigation with other non-WJ IV measures.

#### 5.1.2. Possible Intermediate Cognitive Ability or Processing Dimensions

An underlying System 1-System II cognitive processing dimension of broad CHC-categorized abilities is suggested by the PNA complimentary MDS + MST analysis (see Figure 3). The *X*-axis finds the left-half populated by Gs tests (Letter–Patten Matching, Number Pattern Matching, Pair Cancellation) and Gr tests (Retrieval Fluency, Phonological Processing–Word Fluency), measures sharing a speed-fluency requirement. The next most adjacent tests are Verbal Attention and Object–Number Sequencing, both measures of working memory (Gwm) requiring significant attentional control. Collectively the *X*-axis appears anchored on the far left by tests of broad CHC abilities representing parameters of cognitive processing efficiency, particularly the ability to control attention under time constraints (Schneider and McGrew 2012, 2018). The tests towards the right half of the *X*-axis are non-speeded or *level* measures (Carroll 1993) that require more controlled or deliberate cognitive processes. This cognitive processing distinction is based on an extensive history of dual-mode cognitive processing research in cognitive psychology (Barrouillet 2011; De Neys and Pennycook 2019; Kahneman 2011) and was previously suggested, in the context of CHC theory, by Schneider and McGrew (2012).

The second dimension (*Y*-axis in Figure 3) is hypothesized to order WJ IV tests from top-to-bottom as per Cattell’s *g_f_* or Ackerman’s *intelligence-as-process* to Cattell’s *g_c_* or Ackerman’s *intelligence-as-knowledge* (particularly auditory–linguistic based knowledge) theoretical distinctions (Ackerman 1996, 2018; Cattell 1943). The *Y*-axis in Figure 3 finds the top half populated exclusively by Gv (Block Rotation, Spatial Relations) and Gf tests (Analysis–Synthesis, Concept Formation), measures requiring more novel “on the spot” processing of nonverbal visual–spatial stimuli. Gf and Gv factors and measures frequently form a single Gf/Gv dimension (Carroll 1993), a construct consistent with Cattell’s notion of a general fluid intelligence ability (*g_f_*). Even though speeded, the next three tests nearest the top of the *Y*-axis are three Gs tests, all which require the processing of visual-spatial or geometric symbols. Conversely, the tests anchoring the bottom of the Y-axes are measures of acquired general verbal knowledge (General Information), comprehension of oral language (Oral Comprehension), and lexical knowledge (Oral Vocabulary). The two Gr tests (Retrieval Fluency, Phonological Processing-Word Fluency) both require fluent retrieval of acquired lexical knowledge. Finally, the Verbal Attention Gwm test requires the processing of verbally presented words and numbers.

#### 5.1.3. Is Cognitive Processing Efficiency or Attentional Control the Key Component of Intelligence?

Compared to common cause factor analysis of intelligence tests, a primary benefit of PNA is its exploratory and descriptive focus that facilitates the development and evaluation of psychological theories (Haslbeck et al. 2021). In the absence of clear guidance for the interpretation of PNA results with cognitive tests, the interpretations offered below should be viewed as well-reasoned research-based hypotheses needing additional study.

Three of the four most central tests (Letter–Pattern Matching-Gs, Verbal Attention-Gwm, Retrieval Fluency-Gr come from the System I (more automatic or automatized cognitive processing) quadrants in the primary CHC network model (see Figure 3). The underlying broad CHC abilities (Gs, Gwm, Gr) have been classified as representing parameters of cognitive processing efficiency (Schneider and McGrew 2012, 2018). Gwm and Gs feature attentional control (AC) as a core cognitive component. Gwm not only reflects how much information can be simultaneously maintained in an active state, but also the efficacy of AC processes (i.e., divided attention, selective attention, and concentration). Gs tests reflect the speed at which attention can be accurately and fluently directed at tasks during task completion (i.e., attentional fluency). The complex of Gwm and AC constructs collectively has been referred to as the *working memory–attentional control complex* (hereafter referred to as Gwm-AC; Hunt 2011) and, more recently, as simply AC (Burgoyne et al. 2022).

Whatever the terms, be they working memory, retrieval fluency, attentional control, cognitive control, executive functioning, top-down control processes, executive attention, processing speed, etc., the extant broad CHC abilities SEM research consistently suggests that the CHC parameters of cognitive processing or Gwm-AC efficiency are crucial to higher-level cognition typically operationally defined as psychometric *g* or Gf (De Alwis et al. 2014; Demetriou et al. 2014; Fry and Hale 1996; Hunt 2011; Kail 2007; Kyllonen and Christal 1990; McGrew 2005; Neubeck et al. 2022; Schneider and McGrew 2018; Tourva and Spanoudis 2020; Unsworth et al. 2021a, 2021b). The Gwm and AC-related constructs have also demonstrated a central role in other areas of brain network research, such as mind wandering (Bressler and Menon 2010; Kane and McVay 2012; McVay and Kane 2012; Smallwood 2010) and focused attention meditation (Lutz et al. 2008; Sedlmeier et al. 2012). These conceptually-related lines of research have demonstrated a link between measures and constructs of cognitive processing efficiency (particularly Gwm, AC, and Gs) and brain network-based models of neural efficiency (Bressler and Menon 2010). This link is also featured in the dynamic mutualism and wired intelligence models of intelligence that suggests working memory capacity may be a “central” cognitive variable or process underlying intelligence. The process overlap theory of intelligence also features multiple domain–general executive functioning, AC and Gwm-related cognitive processes in the positing of a central executive bottleneck processing explanation of psychometric *g* as an emergent property (Conway et al. 2021; Conway and Kovacs 2015). Engle and colleagues’ (Burgoyne et al. 2022) AC explanation of the positive manifold is also consistent with the importance of the Gwm-AC complex.

#### 5.1.4. Why Is Oral Comprehension Most Central in the CHC Network Model?

The WJ IV Oral Comprehension measure, a CHC-classified measure of listening ability (Gc-LS), was the most central measure in the primary CHC network. This finding was unexpected. Thus, a closer look at the task requirements and PNA results were required.

The “Oral Comprehension is a test of oral language measuring the ability to comprehend a short audio-recorded passage and then supply the missing word using syntactic and semantic cues. This oral cloze procedure requires the use of listening, reasoning, and vocabulary abilities (Mather and Wendling 2014, p. 12).” Schneider (2016) hypothesized that the listening-based cloze procedure may require an examinee to make predictive inferences—“for some theorists, making predictive inferences is one of the brain’s primary functions….a predictive inference is when a person anticipates what a speaker (or author) is about to say. For example, ‘My opponent and I disagree about many things, but we both want what is______ for our country’“ (p. 194). These descriptions suggest that performance on Oral Comprehension is related to multiple CHC abilities.

A review of the primary network model weights matrix found Oral Comprehension having its highest edge weights with measures of Gc/Gf (Verbal Analogies, .23), Gc (Oral Vocabulary, .22), Gs (Letter–Pattern and Number–Pattern Matching, .19 and .15), Gr (Retrieval Fluency, .16), Gv (Block Rotation, .15), and Gwm (Verbal Attention, .13). Yet, the complimentary MDS + MST model (Figure 3) suggests that of these seven measures, Oral Comprehension is most associated (based on proximity) with Verbal Analogies, Oral Vocabulary, Verbal Attention, and Retrieval Fluency. Collectively, these results suggest that performance on measures of oral or listening comprehension is due to the complex interaction of cognitive (working memory, attention, inference, theory of mind, comprehension monitoring) and foundational language abilities (Kim 2016; Osada 2004). Given the complexity and uncertainty of explanations for the central role of the Oral Comprehension measure, this measure of listening ability requires additional research before placing the findings in the context of a theory of intelligence or in suggesting possible intervention strategies. The Oral Comprehension measure, to some degree, requires the cognitive efficiency of the Gwm-AC complex previously described.

### 5.2. The Implication of a CHC Network Model of Intelligence for Interventions

The majority (3 of 4) of the most central measures in the primary CHC intelligence network represent indicators of general cognitive processing efficiency or the Gwm-AC complex. The fourth measure (Oral Comprehension) also requires these abilities. Thus, the Gwm-AC cognitive efficiency constructs are the CHC abilities that may have the greatest probability of influencing overall intellectual or cognitive functioning as defined by a CHC theoretical network. Two of the current authors have suggested (McGrew 2013; Schneider and McGrew 2012, 2013, 2018; Taub et al. 2007) that AC, defined as “the ability to manipulate the spotlight of attention flexibly to focus on task-relevant stimuli and ignore task-irrelevant stimuli….sometimes referred to as spotlight or focal attention, focus, control of attention, executive controlled attention, or executive attention” (Schneider and McGrew 2018, p. 99), may be one of the most central cognitive abilities for understanding the complex and dynamic system of interrelated cognitive abilities expressed as intelligence, and thus, might be an important target for cognitive-based interventions.

As per the centrality hypothesis assumption, “as highly central nodes go, so should go the network” (Robinaugh et al. 2016, p. 748). Accordingly, modification of the central CHC cognitive efficiency processing variables should propagate throughout the cognitive network. This sounds simple, yet, even in areas of psychology that have been studied for over a decade with network models and methods (e.g., psychopathology networks of mental disorders) significant theoretical, experimental, and methodological work still remains (Fried 2020). Adding to the difficulty in translating mental disorder psychopathology PNA research principles to intelligence networks is the disconnect between potential cognitive interventions and those based on clinical treatment mental disorder principles such as *alleviating* or *aggravating* interventions for changing symptoms (Lunansky et al. 2022). We suggest that a different category of “interventions” may be needed to translate intelligence test network findings to applied test interpretation in educational settings. One possible model is Mascolo’s *MARC* model, which is embedded in a *Systematic Method of Analyzing Results for Tailoring Interventions* (SMARTI; Mascolo et al. 2014). The MARC system categorizes cognitive–academic interventions as *modifications* (e.g., changing the content or expectation parameters of what is taught or measured), *accommodations* (e.g., changing conditions under which learning occurs or is measured), *remediations* (e.g., programs designed to ameliorate cognitive and academic deficits), and *compensations* (e.g., interventions intended to bypass, minimize, or compensate for the impact of a cognitive deficit). It is premature to suggest specific PNA-derived CHC intelligence network-based interventions based on the MARC model.

An intriguing recent suggestion, albeit likely controversial in some circles given the historical realities and track record of intelligence testing and theories with marginalized groups, is that newer non-*g* emergent property theories of intelligence might lead to better intervention research for individuals who have been marginalized by society. Holden and Hart (2021) suggest that network-based theories, particularly those that feature Gwm-AC mechanisms (process overlap theory in particular) may hold promise as a vehicle for improving, and not harming, social justice and equity practices and valued outcomes for individuals in marginalized groups. For example, *stereotype threat* (Spencer et al. 2016; Steele and Aronson 1995) has been linked to poorer outcomes in performance settings where an individual’s group membership is salient, a situation that can negatively impact an individual’s Gwm-AC complex, executive functions, and more deliberate controlled cognitive processing mechanisms (Holden and Hart 2021; Spencer et al. 2016). The identification of the central Gwm-AC complex and a possible System I-II cognitive processing dimension in the current study aligns with Holden and Hart’s (2021) proposal that these cognitive constructs should be featured in a variety of potential interventions for learners who experience learning difficulties, and as articulated by Holden and Hart (2021), to potentially mitigate the impact of stereotype threat in certain marginalized groups. In contrast, common cause factor models that include a dominant psychometric or theoretical *g* construct hold little promise for helping individuals as a century of research has not yet found convincing evidence-based practice approaches for “moving the needle” on general intelligence. In contrast, emergent property models have “the benefit of focusing on lower order specific abilities…because they are real, and beyond being statistically emergent (like global IQ or *g*), they have predictive validity” (Holden and Hart 2021, p. 3).

However, a strong caveat is needed. The extant cognitive training literature has failed to provide convincing evidence for cognitive enhancement programs. The current findings reinforce the contemporary focus of brain or neuroscience-based cognitive training programs for improving such cognitive efficiency abilities as Gwm-AC and executive functioning processes. These may be the cognitive levers most likely to impact overall cognitive performance. However, at best, reviews of brain- or neuroscience-based cognitive training programs have only suggested “guarded optimism” (Green and Newcombe 2020). The bulk of the research evidence indicates that although such programs can produce near transfer to tasks like the training tasks, far transfer generalization has not been demonstrated to the extent these programs are ready for widespread implementation (Cowan 2014; Green and Newcombe 2020; Jaeggi et al. 2011; Shipstead et al. 2012). The “brain training” literature is currently controversial (see Simons et al. 2016 for a review of the recent controversies and conflicting positions of groups of experts). The current results should not be interpreted as an endorsement of brain training programs to improve educational outcomes. The current results only suggest that cognitive efficiency-focused training programs may hold the best promise for improving cognitive performance, and indirectly, academic outcomes of learners.

### 5.3. Implications for Intelligence Testing Interpretation

The results of this study have implications for the interpretation of broad CHC scores in intelligence testing in general and select WJ IV tests and clusters in particular.

#### 5.3.1. Interpretation of Broad CHC Scores in Intelligence Testing

Practitioners must make sense of the particulars of individual observations. Theory-based models help us distinguish between variations that are predictable and those that are coincidental.

Latent variable factor analysis intelligence models give practitioners tools for understanding which sets of tests tend to move together in person-level fluctuations. For example, it is a good guess that when a person has high scores on several tests of the same latent variable, the high scores result from a high value on the latent variable. For this reason, aggregating these test scores into broad CHC ability composites makes sense.

However, any practitioner can tell you that test scores do not always fluctuate in ways that are easy to interpret in terms of a model. In latent variable models with simple structure, test indicators of the same latent variable might differ because of test-specific factors (measurement error and reliable specific factors). If test indicators of different latent variables are both high or both low, the reason is attributed to coincidence.

In PNA test models, test scores still fluctuate at random, but some pairs of test scores are more likely to move in the same direction than others. The network’s edge weights are partial correlation coefficients, which quantify the relation between test score pairs after controlling for all other tests in the model. If two tests with near-zero partial correlations are both profile outliers in the same direction, then luck or random factors are generally the best explanations. If two tests with strong partial correlations are both outliers in the same direction, luck may have played a role in the score fluctuations, but it may be profitable to consider other possibilities. Perhaps further exploration and testing is warranted to identify narrow strengths or weaknesses that are replicable and consequential to the individual.

PNA intelligence models do not just inform us about the independent pairwise relations among tests. They encourage us to think about broad CHC abilities in a broader way. Latent variable models channel our thoughts toward thinking of general, broad, and narrow latent ability constructs as distinct entities that differ in terms of their generality but are essentially the same type of entity—they are abilities that exert influence on test performance. It is not necessarily wrong to think of abilities in this way, but a broader range of possibilities remains open (Schneider et al. 2016).

If a network model account of broad CHC ability tests is correct, broad CHC ability test may function less like an officer directing soldiers and more like a colony of ants in which control emerges from the collective exchange of information of each ant with the other (Gordon 2010). That is, broad CHC abilities might not be latent abilities directing narrow abilities and tests to move this way and that. Instead, they emerge from the interplay of many brain modules exchanging information. What then, does a broad CHC ability score represent? It does not represent an estimate of a single ability but is instead a summary statistic much like GPA is not an estimate of a latent grade lurking behind observable grades but is instead a summary of academic performance over time. This subtle shift in perspective orients practitioners away from thinking about broad CHC abilities as siloed entities and toward viewing them as interconnected causal systems with multiple paths by which tasks and test performance are accomplished.

#### 5.3.2. Implications for Interpretation of Select WJ IV Tests and Clusters

The selection of CHC measures was driven by the goal of using the best CHC theory-relevant WJ IV measures possible in a CHC-focused PNA. The goal was not to evaluate the validity of the WJ IV CHC cluster measures per se. Nevertheless, the results can inform WJ IV users of the strengths and limitations of select WJ IV measures and clusters.

The robustness of the WJ IV broad CHC clusters of Comprehension–Knowledge (Gc; Oral Vocabulary, General Information) and Cognitive Processing Speed (Gs; Letter–Pattern Matching, Pair Cancellation) clusters was confirmed. The a priori decision to exclude certain WJ IV COG cluster tests (see Supplementary Materials Section S1) limited a thorough evaluation of the validity of other clusters. The three Short-term Working Memory Gwm tests (Verbal Attention, Object–Number Sequencing, and Memory for Words) were found to be consistent measures of the Gwm domain. The Numbers Reversed measure was deliberately absent. However, given the lengthy history of digit reversal tasks being valid indicators of working memory in other IQ tests, we judge the WJ IV Short-term Working Memory cluster to be a valid measure of Gwm (also see Schneider 2016). Although the WJ IV Visual Processing cluster Picture Recognition test was deliberately absent, a tight Gv domain was identified by the Spatial Relations and Block Rotation test measures. This result suggests that Schneider’s (2016) conclusion that the previous WJ III cluster (combination of the full-length Spatial Relations and Block Rotation tests) should have been retained as a valid cluster instead of the WJ IV “shrinking” each measure to subtests and combining them as a single test measure. Given that the WJ IV Visualization test and Visual Processing clusters have similar reliabilities across all ages in the WJ IV norm sample (.85 and .86. respectively), the single Visualization test could serve as a single indicator of the broad CHC Gv ability.

A valid WJ IV COG Auditory Processing cluster (Ga; Phonological Processing; Nonword Repetition) cluster had no chance of emerging since the WJ IV Phonological Processing test was replaced by its three subtests and Nonword Repetition was excluded. Instead, a Ga ability dimension emerged, defined by two Phonological Processing test measures (Word Access; Word Substitution) together with the OL Segmentation and Sound Blending measures. As expected, based on post-publication analyses and reviews (Schneider 2016), the Phonological Processing Word Fluency measure, which requires examinees to tell the examiner as many words as they can that start with a particular sound (within a one-minute interval), is a close relative of the Retrieval Fluency measure in task demands (name as many types of certain objects within a minute). The Retrieval Fluency and Phonological Processing–Word Fluency measures formed a dyad representing the broad CHC retrieval fluency (Gr) ability. Thus, users of the COG Auditory Processing (Ga) cluster should cautiously interpret this composite score as a mixture of at least three broad CHC abilities—Ga, Gwm, and Gr. The Phonetic Coding (Ga) cluster from the OL battery, which is comprised of the Segmentation and Sound Blending measures, is likely a better WJ IV Ga cluster. As suggested by Schneider and McGrew (2018), for current users, the best available and empirically supported proxy for Gr within the WJ IV battery is the OL Speed of Lexical Access cluster. Finally, as discussed in the context of intelligence theory implications, when the two WJ IV Gl measures were included in the 25-measure secondary sensitivity analysis, their exclusion was justified. This raises questions about the validity of the WJ IV Gl cluster in clinical test interpretation.

The Number Series measure was not included in the primary analysis (Figure 1 and Figure 3). In the 23-measure complimentary model MDS + MST figure (Figure 4), Number Series was directly connected with the third WJ IV Gf measure—Analysis–Synthesis, a controlled learning miniature math logic system. Analysis–Synthesis was, in turn, connected with the second Gf cluster measure, Concept Formation. The proximity of these three Gf measures supports the validity of the WJ IV Gf cluster. However, in Figure 4, Number Series was closer to, and formed a cohesive community with, two Gq math achievement measures (Calculation; Applied Problems). This suggests that although number series tasks may historically be one of the best indicators of Gf (Carroll 1993), the WJ IV Number Series measure might be unnecessarily confounded with foundational math procedural or declarative achievement knowledge. When evaluating school-aged individuals experiencing problems with mathematics, the WJ IV Fluid Reasoning cluster should be interpreted with caution. If the Number Series measure is significantly lower than the Concept Formation measure, examiners should examine performance on measures of quantitative-based deductive reasoning, purged of math achievement (i.e., Analysis–Synthesis).

### 5.4. Methodological Implications for the PNA Investigation of Intelligence Tests

The results of the current study reinforce the PNA methodological cautions and recommendations of Jones et al. (2018), Letina et al. (2019), and Neal and Neal (2021). First, the benefits of supplementing multidimensional PNA models with lower-dimension data visualization methods (e.g., variants of MDS and MST) are supported. Second, these complementary data visualization methods revealed potentially important and unexpected insights regarding two higher-order cognitive ability or processing dimensions for CHC theory and measure interpretation. Third, the “what if” sensitivity boundary specification models facilitated an analysis of the a priori specified CHC model and variable selection criteria. The sensitivity analyses also enhanced the evaluation of the robustness of the primary CHC model. Fourth, evaluating the accuracy and stability of the CHC theoretical model was demonstrated through case-dropping subset bootstrap methods. Finally, the primary and secondary sensitivity analysis reinforced the importance of using a well-developed theoretical psychological framework (e.g., CHC theory) to guide the selection of construct indicators in PNA psychological research. 

PNA no-*g* model findings compliment, but do not supplant, traditional common cause factor analysis models. PNA is recommended to be added to the traditional common cause factor analysis studies of IQ batteries. PNA should be included in all future IQ test manuals, as well as research by independent scholars. Less parochial and method-specific IQ test structural research could reduce piecemeal publications that are often not synthesized for applied assessment personnel. Such an ecumenical approach would require researchers to present results from the major classes of IQ test structural research methods (including PNA) and clearly articulate the theoretical basis for the model(s) the author’s support. Such an approach would also gently nudge IQ test structural researchers to minimize the frequent conflation of theoretical and psychometric *g* constructs. Such multiple-methods research in test manuals and journal publications can better inform users of the strengths and limitations of IQ test interpretations based on whatever conceptualization of psychometric general intelligence (including models with no such construct) underlies each type of dimensional analysis.

## 6. Limitations and Future Directions

The scope and complexity of the current study results in several limitations. We highlight five general limitations and offer suggestions for new research.

First, the current study is formative in nature. As far as we know, this study is the first attempt to go beyond the generation of an IQ test global PNA model to the generation of practical IQ test interpretations as per contemporary theories of cognitive processing and intelligence. The methods and operational decisions made in the current study need additional study in other samples and with other IQ batteries and theory-based collections of CHC measures.

Second, the broad-stroke nature of the current study leaves many questions unanswered. Additional studies are needed to ascertain which components of cognitive efficiency or the Gwm-AC complex (i.e., AC; retrieval processes; different executive function processes) may be most crucial in the CHC intelligence network, what models, measures, and research paradigms should be used to validate the hypothesized AC variance in individual tests (von Bastian et al. 2020), and the direction of effects between and among these related constructs. Moreover, similar CHC-grounded PNA studies should examine whether the Gwm-AC complex findings are invariant across important demographic characteristics (e.g., gender, race, cultures, etc.). Additionally, the unexpected centrality of the WJ IV Oral Comprehension measure, a CHC Gc measure of listening ability, needs further examination.

Third, the generation of possible cognitive interventions, based on network centrality metrics and network science principles, is an advantage of PNA methods over traditional *g*-centric and mixed-*g* common cause factor analysis models and related SEM causal model research. Yet, this is currently unrealized “potential.” Despite promising no-*g* and mixed-*g* CHC broad cognitive–achievement relations research findings, an evidence-based direct link between CHC test score-based interpretations and cognitive or academic interventions and diagnostic practices remain elusive and controversial. We urge assessment professionals to resist the temptation to immediately attach practical implications to cognitive efficiency and listening comprehension measures as being diagnostic or directly relevant to interventions, as suggested by the current study.

A distinct advantage of bridging PNA models to potential interventions should be pursued by researchers—namely, conducting *in silico* mathematical and computational simulations to evaluate various theories and models and network relations (Haslbeck et al. 2021; Lunansky et al. 2022) Computer-based network simulation models allow “one to deduce what would happen upon changing the connectivity of the system” and thus, the ability to explore possible causal pathways that can propagate to other nodes in the network (Epskamp et al. 2018b, p. 976). If a robust CHC cognitive network model can be replicated, the impact of node-specific perturbations of the most central nodes can be modeled mathematically and computationally to ascertain the projected impact on other nodes in the CHC network. Such computer simulations could study the potential impact of interventions focused on the most central CHC measures and underlying abilities.

An important next step would be a network analysis of cognitive and achievement domains linked to theory (e.g., see Angelelli et al. 2021; Zoccolotti et al. 2021). We are currently unfamiliar with methodological options for exploring relations between a set of network predictor measures (cognitive) with a set of network outcome measures (achievement) in the same network, or linked sets of networks. The descriptive, exploratory, and theory-generating strengths of PNA models can inform the specification and testing of SEM causal models that include both CHC cognitive and achievement variables. Such integrated cognitive and achievement PNA studies could provide new information that can contribute to the resolution of several of the salient issues in the ongoing debates regarding the use of the pattern of strengths and weaknesses method for SLD identification.

Finally, pursuing the development of formal theories of the complex interaction of cognitive variables that produce general intellectual functioning, which would also include achievement outcomes, is a daunting task. It will require a rigorous multi-stage process of moving from simple descriptive models (like the one presented in this paper) to more formal theories that produce comparisons of “empirical data models to theory-implied data models in order to evaluate and refine an existing formal theory” (Haslbeck et al. 2021, p. 1). We believe Haslbeck et al.’s (2021) proposed framework for advancing theory construction in psychopathology could be adapted for intelligence research. In this framework, formal theory construction will likely require a division of labor between researchers steeped in intelligence test measurement, psychometrics, and psychometric-derived intelligence descriptive taxonomies (e.g., CHC theory) and intelligence or cognitive science theoretical researchers who can focus more on the generation, evaluation, and refinement of formal theories of intelligence and cognitive functioning. Clearly, the lengthy historical chasm between proposed intelligence testing score diagnostic and interpretation systems and evidence-based interventions will likely persist until a genuine rapprochement occurs between these two general categories of intelligence researchers. We hope the current paper serves as a demonstration of how newer network-based intelligence theories and analytic methods might “jump-start” these collaborative efforts, efforts devoted to an eventual understanding of the complex, non-linear, dynamic nature of human intelligence and its measurement.

## 7. Concluding Comments

“All models are wrong, but some are useful” (Box and Draper 2007, p. 414). Just as we believe the strong *g*-centric position of the Canivez et al. IQ test research group is largely due to certain methodological assumptions and a restrictive type of factor analysis, and the mixed-*g* Keith–Reynolds studies are also influenced by the presence of a prominent hierarchical psychometric *g*-factor, the current PNA-based results should only be considered one of multiple conceptual and methodological lenses from which IQ test structural research should be examined. Intelligence testing structural researchers need to recognize the fact that different common cause factor analytic and newer PNA models all provide statistically defensible accounts of the positive manifold of a collection of IQ subtests. No single statistical structural model can claim preeminence. Theoretical considerations need to play a significant role when comparing results derived from statistical models based on different assumptions that, in turn, shape and constrain the final structural solution (Fried 2020).

The current dominant *g*-centric message of “just say no” to the interpretation of IQ test broad CHC scores has claimed a premature victory as a proven evidence-based practice recommendation. The current no-*g* PNA research and modern network intelligence theories, together with the extant no-*g* or mixed-*g* CHC cognitive–achievement relations research, support the relevance of CHC broad ability scores for understanding school achievement. The current findings also suggest that newer emergent property theories of intelligence and associated network analysis methods should command more serious attention among IQ test structural researchers.

## Figures and Tables

**Table 1:** Gender and race demographics of WJ IV ages 9–19 norm sample used in the current study.

Gender	White	Black	Indigenous	Asian/Pacific Islander	Other
Male	1261 (38.7%)	214 (6.6%)	9 (0.3%)	73 (2.2%)	41 (1.3%)
Female	1265 (38.8%)	274 (8.4%)	14 (0.4%)	74 (2.3%)	33 (1.0%)

**Table 2:** 20-measure primary and 23-measure secondary sensitivity network centrality measure metrics.

		Network Relative Centrality Characteristic Metrics					
		20 Measure Primary Model			23 Measure Sensitivity Model		
WJ IV Measure	CHC Domain	Between.	Close.	Strength	Between.	Close.	Strength
Analysis-Synthesis	Gf	0.35	0.84	0.82	0.12	0.77	0.73
Concept Formation	Gf	0.29	0.80	0.65	0.06	0.71	0.64
							
Verbal Analogies	Gc/Gf	**0.94**	0.87	0.84	0.32	0.84	0.76
General Information	Gc	0.12	0.73	0.65	0.00	0.66	0.64
Oral Comprehension	Gc	**1.00**	**1.00**	**0.98**	**1.00**	**1.00**	**1.00**
Oral Vocabulary	Gc	0.88	0.82	0.88	0.47	0.75	0.81
							
Block Rotation	Gv	0.53	0.90	0.90	0.47	**0.90**	0.89
Spatial Relations	Gv	0.12	0.82	0.76	0.06	0.82	0.67
							
Phon. Proc.-Word Access	Ga	0.41	0.79	0.77	0.15	0.74	0.67
Phon. Proc.-Substitution	Ga	0.65	0.79	0.87	0.47	0.80	0.83
Segmentation	Ga	0.18	0.73	0.69	0.03	0.71	0.62
Sound Blending	Ga	0.35	0.78	0.64	0.21	0.77	0.66
							
Phon. Proc.-Word Fluency	Gr	0.24	0.87	0.78	0.06	0.79	0.74
Retrieval Fluency	Gr	0.65	**0.92**	**0.96**	0.35	0.86	**0.92**
							
Object-Number Seq.	Gwm	0.41	0.86	0.73	0.21	0.82	0.64
Memory for Words	Gwm	0.29	0.81	0.79	0.12	0.73	0.75
Verbal Attention	Gwm	0.77	**0.95**	**0.97**	0.47	0.85	0.88
							
Letter-Pattern Matching	Gs	**1.00**	**0.93**	**1.00**	**0.68**	**0.92**	**0.94**
Number-Pattern Matching	Gs	0.18	0.85	0.86	0.32	0.88	0.90
Pair Cancellation	Gs	0.18	0.81	0.67	0.18	0.83	0.71
							
Number Series	Gq				0.44	0.82	**0.96**
Applied Problems	Gq				0.18	0.85	0.81
Calculation	Gq				0.15	0.82	0.73

Note. Bold font designates the top three (sometimes four) relative centrality values in each column. The one exception is the betweenness column for the 23-variable analysis model where the second highest value was only .68 for Letter–Pattern Matching.

**Table 3:** Correlations between PNA-relative centrality metrics and psychometric *g*-loading information for 20 primary measures in 20- and 23-measure primary and secondary analysis models.

	23-Variable Sensitivity Analysis Model			
20 Variable Primary Model	Betweenness	Closeness	Strength	*g*-Loading (PCA)
Betweenness	.84	.58	.69	.43
Closeness	.77	.88	.81	−.05
Strength	.81	.74	.93	.02
*g*-loading (PCA)	.10	−.24	−.15	

## Data Availability

Data used in this study are the proprietary property of Riverside Insights (formerly, Riverside Publishing). All data are solely owned and licensed by Riverside Insights and thus cannot be shared by the authors in any form or format. Requests to access the data should be directed to Riverside Insights.

## Notes

1 The intelligence “test” and CHC factor and theory literature can be confusing. There are IQ test scores, individual subtests, individual tests, CHC factors, abilities, scores, etc. In this paper, the following terms and definitions are used. The commonly referred to “subtests” (e.g., Wechsler subtests) are referred to as individual tests, or just tests. *Tests* are the smallest individual measure that provides obtained scores for interpretation. Some tests may be comprised of *subtests*, that are mini-tests for which no derived score is provided. Subtests are typically combined to form a test (e.g., WJ IV General Information test consists of the “what” and “where” subtests). Two or more tests are typically combined into composite or index scores (e.g., Wechsler Processing Speed Index) that represent broad stratum II abilities as per CHC theory (e.g., Gs). These are called *broad CHC scores*. Broad CHC stratum II theoretical constructs are called *broad CHC abilities*. The combination of tests that provide a total composite IQ score representing general intelligence (*g*) is called the *global IQ*. A self-contained published battery of tests is called an *IQ battery*, not an IQ test.

2 Henceforth in this paper, for economy of writing, in the analysis and results sections the different WJ IV tests or subtests are referred to as the WJ IV measures. *Measures* designates the WJ IV test and subtest scores used in the PNA analysis.

3 It is often misunderstood that Horn and Carroll’s broad Gf and Gc abilities are not isomorphic with Cattell’s two *g_f_/g_c_* general capacities, constructs that are more consistent with the notion of general intelligence (*g*) as articulated by Cattell’s mentor, Spearman (see Schneider and McGrew 2018). The *g_f_/g_c_* notation used here refers to Cattell’s two general types of intelligences. Ackerman’s complete PPIK theory of intelligence includes the broad constructs of intelligence-as-process, personality, interests, and intelligence-as-knowledge.

## Supplementary Materials

The following supporting information can be downloaded at: https://www.mdpi.com/article/10.3390/jintelligence11010019/s1, Section S1, Systematic CHC-model Based Process Used to Select Measures for Analysis; Section S2, Creation of More Interpretable Versions of the Network Figure 1 and Figure 2 in Main Article Text, Figure S1: Weighted undirected network structure of 20 select WJ IV measures of seven broad CHC abilities in the primary network model, Figure S2: Weighted undirected network structure of 23 select WJ IV measures of eight broad CHC abilities in the secondary sensitivity network model; Section S3 , Quantile Distribution of 20-measure Primary CHC Network Model, Figure S3: Quantile distribution of network edge weights from primary network model (Figure 1 and Figure S1); Section S4, Network Centrality Metrics Case-dropping Subset Bootstrap Stability Analysis, Figure S4: Case-dropping Bootstrap (*n* = 1000) Correlation Stability (CS) Coefficients for 20-measure CHC Network Model Centrality Indices; Section S5, 25-measure (inclusive of Gl Measures) Sensitivity Analysis Results, Figure S5: Weighted undirected network structure of 25 select WJ IV measures of nine broad CHC abilities in secondary sensitivity network model, Figure S6: 2-D multidimensional scaling analysis (Guttman Radex) of 25-measure secondary sensitivity model connected by minimal spanning tree algorithm; Section S6, Psychometric *g*-loadings for 20 Primary Measures, Table S1: Psychometric *g*-loadings for 20 primary measures; Section S7, Post-hoc 19-measure (Oral Comprehension excluded) Sensitivity Model Analysis, Figure S7: Weighted undirected network structure of 19 (Oral Comprehension excluded) select WJ IV measures of seven broad CHC abilities in primary network model, Table S2: 19-measure (Oral Comprehension excluded) primary sensitivity network model centrality measure metrics. (Comrey 1988), (Epskamp et al. 2012), (McGrew and Flanagan 1998), (Messick 1995), (Pedersen 2022a, 2022b), (Wickham 2016) and (Wilke 2020) are cited in the supplementary materials.
