# Supplementary material for: A Psychometric Network Analysis of CHC Intelligence Measures: Implications for Research, Theory, and Interpretation of Broad CHC Scores “Beyond g”
Source: J Intell. 2023 Jan 16;11(1):19. doi: 10.3390/jintelligence11010019 (PMC9865475; doi:10.3390/jintelligence11010019)
Supplement: Supplementary file 1 [file jintelligence-11-00019-s001.zip › jintelligence-2008396-supplementary.pdf]

Journal of
Intelligence

MDPI

Supplementary Materials

A Psychometric Network Analysis of CHC Intelligence Measures: Implications for Research, Theory, and Interpretation of Broad CHC Scores "Beyond *g*"

Kevin S. McGrew [1,*], W. Joel Schneider [2], Scott L. Decker [3] and Okan Bulut [4]

[1] Institute for Applied Psychometrics, 1313 Pondview Lane E, St. Joseph, MN 56374, USA
[2] College of Education and Human Development, Temple University, Ritter Hall 358, Philadelphia, PA 19122, USA
[3] Applied Cognitive Neuropsychology Lab, Department of Psychology, University of South Carolina, Columbia, SC 29208, USA
[4] Centre for Research in Applied Measurement and Evaluation, University of Alberta, Edmonton, AB T6G 2G5, Canada
* Correspondence: iqmcgrew@gmail.com

Section S1: Systematic CHC-model Based Process Used to Select Measures for Analysis

Using a set of multiple criteria, a systematic and deliberate process was followed to select the best possible CHC construct measures to minimize potential boundary specification issues (Bringmann et al. 2019; Neal and Neal, 2021). The process is described below.

First, the artificial boundaries of the four separate WJ IV batteries (COG, OL, ACH, and ECAD) were ignored in the selection of tests and subtests.[1] This provided the largest pool for selecting measures to best define broad CHC ability domains. Second, the extant structural analysis research of the last three versions of the WJ battery were reviewed (i.e., confirmatory factor analysis of the WJ-R, WJ III and WJ IV; exploratory cluster, factor, and multidimensional scaling analysis of the WJ IV; hereafter this body of research is referred to as the *extant WJ-R to WJ IV CHC research*) as per contemporary CHC theory (Schneider and McGrew 2018). The measures were selected not to conform to the CHC cluster composition in the published WJ IV, but rather, to provide the most empirically valid and "pure" indicators of CHC broad cognitive ability theoretical constructs. Third, post-WJ IV publication, significant revisions were made to the CHC taxonomy as well as cogent

[1] Most psycho-educational test batteries refer to the individual measures in the battery as subtests or tests. In the WJ IV battery, some tests are comprised of two or more "subtests," which are, in effect, brief mini-tests that do not produce individual standardized scores. For example, the COG General Information test is comprised of the separate General Information-What and General Information-Where subtests. The OL language tests are measures of Ga, Gwm, Gc, and Gs cognitive domains in CHC theory (see McGrew et al. 2014). Except for two math achievement tests (Calculation and Applied Problems) included in a second supplementary sensitivity analysis, tests were not selected from the WJ IV Tests of Achievement (ACH). One test (Verbal Analogies) from the age-limited 10 test co-normed WJ IV Early Cognitive and Academic Development (WJ IV ECAD; Schrank et al. 2015) battery was also used. Although the ECAD is focused on preschool and early school years (2 through 7 years of age), norm data was gathered for all 10 ECAD tests through late adulthood as per the WJ IV (McGrew et al. 2014). The complete age-range Verbal Analogies test was used given the historical prominence of verbal analogies tests in intelligence research and its factorially complex loading on the two most important CHC broad abilities—Gf and Gc.

criticisms were made of some of the WJ IV cognitive measures. In particular, the CHC Glr (long-term storage and retrieval) ability was split in to separate Gl (learning efficiency) and Gr (retrieval fluency) abilities (Schneider and McGrew 2018). Schneider and McGrew (2018) suggested that when using the WJ IV batteries, the WJ IV COG Long-term Storage and Retrieval cluster should be considered a measure of Gl and the OL Speed of Lexical Access cluster be interpreted as measuring Gr. Despite the Gl recommendation, the extant WJ-R to WJ IV CHC research has not provided robust convincing support for the pairing of the Visual-Auditory Learning and Story Recall tests as a strong Gl composite. This is consistent with Schneider's (2016) criticism of the Visual-Auditory Learning test as not being a strong indicator of associative memory (MA) as per CHC theory. These two Gl tests were thus excluded from the primary and first secondary sensitivity analyses. Furthermore, Schneider (2016) criticized certain WJ IV subtest-based measures (e.g., the COG Phonological Processing test is comprised of three subtests) for producing tests that are mixed measures of multiple CHC abilities (i.e., "Frankentests"). To select the most valid pool of CHC broad ability construct indicators from the complete WJ IV battery, where appropriate, select subtests replaced the composite tests they comprised. The Word Access, Word Substitution, Word Fluency subtests were used instead of the single Phonological Processing test.[2]

The overriding goal of the careful measure selection process was to operationalize each CHC broad ability domain with three of the most valid WJ IV CHC ability domain indicators. This goal was achieved for all but the CHC domains of Gl and Gv. The WJ IV Gv cluster Picture Recognition measure was omitted based on Schneider and McGrew's (2018) agreement with Carroll (1993) who stated that he was not aware of "any research on the usefulness of visual memory tasks in predicting educational or occupational success" (p. 284). Only two robust valid indicators were identified for Gv (i.e., the Visualization measures of Spatial Relations and Block Rotation that correlate .54 in the current sample). Some measures that may have been featured in certain CHC-based WJ IV published clusters were not selected when the extant WJ-R to WJ IV CHC research suggested a measure had mixed CHC construct variance. For example, Numbers Reversed has been reported to measure other secondary CHC variance. The Numbers Reversed test displayed primary Gwm loadings and secondary Gq loadings in the WJ IV norm data, with the secondary Gq factor loading most likely representing quantitative stimulus content characteristics (McGrew et al., 2014).

As per the CHC test construction and interpretation principal of including multiple qualitatively different narrow ability measures in a composite score to maximize adequate construct representation (Comrey 1988; Messick 1995; McGrew and Flanagan, 1998), some CHC domain measures were selected over others (in the same domain) to provide broader coverage of the CHC broad ability. For example, Oral Comprehension, a Gc measure of listening ability (LS) added more unique narrow ability coverage of the Gc domain (as represented by the COG measures of Oral Vocabulary-VL and General Information-K0) than would another indicator of VL (Picture Vocabulary) (McGrew et al. 2014). Number Series, a featured measure in the WJ IV COG, was not used in the primary 20-measure PNA given Schneider's (2016) cogent criticism of the test being neither purely academic nor cognitive but occupying "…a weirdish wild space in between" (p. 196). However, Number Series was included in a 23-measure supplementary sensitivity analysis for three reasons. First, the Number Series measure is featured prominently in the WJ IV COG General Intellectual Ability (GIA) and Gf clusters. As reported in the WJ IV Technical Manual, number series measures have a long history as strong measures of Gf (Carroll 1993) and Number Series was the WJ IV's strongest measure of Gf and one of the strongest indicators of psychometric *g* and cognitively complexity (McGrew et al. 2014). Second, Bulut et al.'s

[2] To reduce confusion, hereafter the individual tests and subtests are called measures.

(2021) PNA of the 14 primary WJ IV COG measures reported Number Series as the most central measure in their reported network, a finding that required further exploration. Third, given Schneider's (2016) criticism that Number Series may be a confounded cognitive (Gf) and achievement (Gq) measure, the impact of including Number Series together with two other Gq math achievement measures (Calculation and Applied Problems) provided the ability to evaluate the stability of the primary 20-measure network model via a secondary 23-measure "what if" boundary specification network sensitivity analysis.[3]

Section S2: Creation of More Interpretable Versions of Network Figures 1 and 2 in Main Article Text

In the main text of this paper, special network model figures (Figures 1 and 2) are presented that also included select salient network edge weights (greater than or equal to .15) and the designation of the most central measure nodes. The raw and lightly edited original network figures (edited in a simple graphic editor to include edge weights and central node designations) produced by the *JASP* software are included below (Figure S.1 and Figure S.2) for comparison purposes. To create the more interpretable network figures in the main text, the following process was used.

The same *R* packages and options that are used by *JASP* were run: *bootnet's* (Epskamp et al. 2018) estimateNetwork function with the *EBICglasso* estimator. As is performed by *JASP*, the graph's node positions were extracted using the *qgraph* package (Epskamp et al. 2012) with the spring layout option and processed with the *tidygraph* package (Pedersen 2022b). The plots were made with the *ggplot2* package (Wickham, 2016) as well as two *ggplot2* extensions, *ggforce* (Pedersen 2022a) and *ggtext* (Wilke 2020).

The reader will note that Figures S1 and S2 are much more visually complex as they include all network edges determined to be significant and reported as per the *JASP* weight matrix. This can be problematic in large samples (current n = 3258) due to the high power of the statistical analysis. As reported in the main text (and illustrated in *Supplementary Material Section 3, Figure S3*), 78% (149 of 190) of the possible edges were identified as being non-zero. Approximately 25% of the edge weights were zero and approximately 50% of the edge weights were zero or were non-zero and equal to or less than 0.05. Including such a large number of negligible edge weights produced the much more complex visual networks in Figures S1 and S2. Thus, to "see the forest from the trees," all trivial edges were "faded out" as per the criterion mentioned above. This produced the less visibly complex and more interpretable Figures 1 and 2 in the main text.

All other supplementary network model figures described presented in this *Supplementary Materials* are presented in the raw unedited *JASP* format.

[3] As per the online *APA Dictionary of Psychology*, sensitivity analysis is "an evaluation of the extent to which the overall outcome of a model or system will be affected by potential changes to the input" https://dictionary.apa.org/sensitivity-analysis. Retrieved 10-27-21.

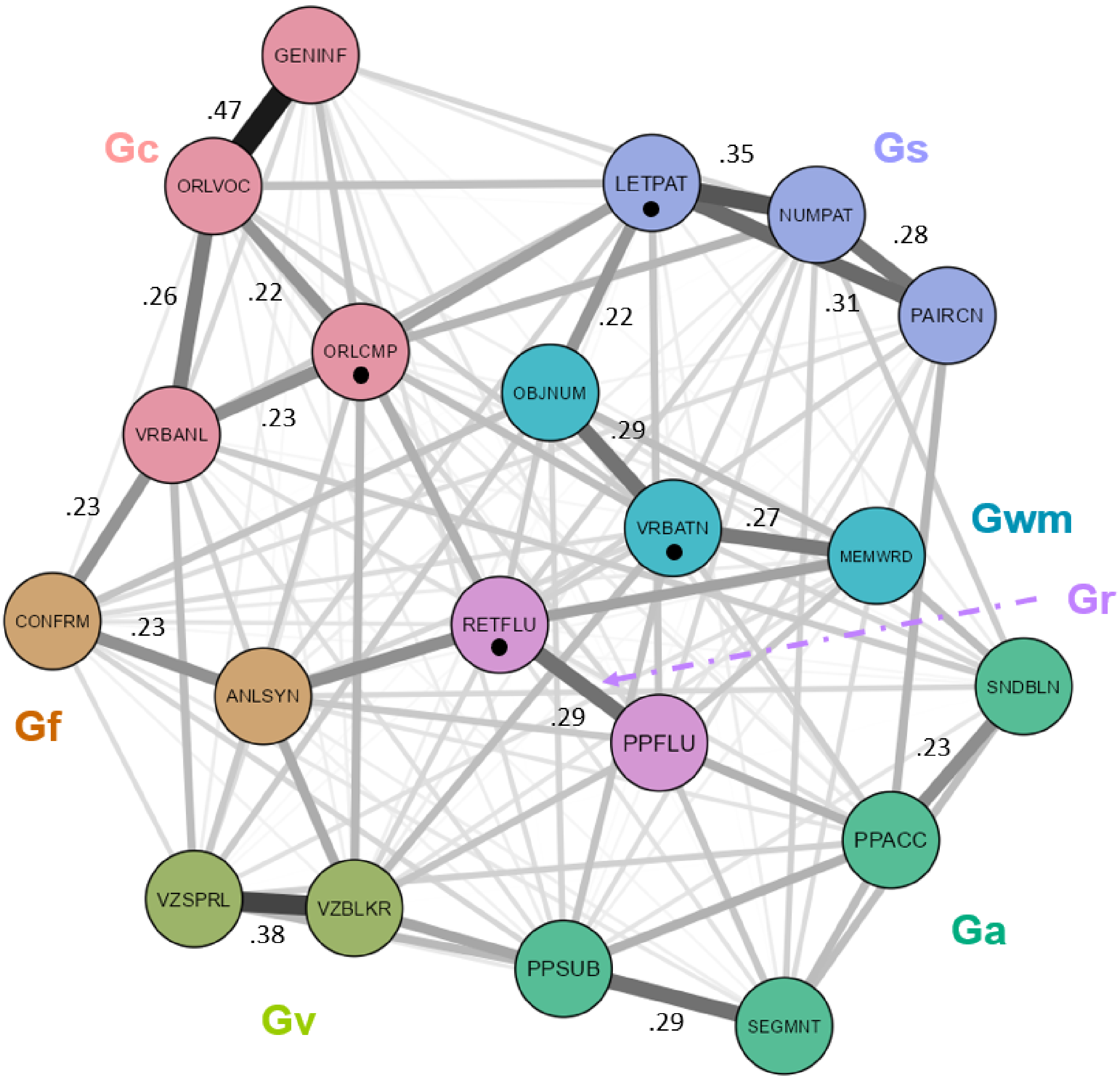

Figure S1. Weighted undirected network structure of 20 select WJ IV measures of seven broad CHC ability domains in the primary network model: JASP produced network model edited to include labels for CHC construct communities, select salient edge weights, and the most central nodes designations (small black circles).

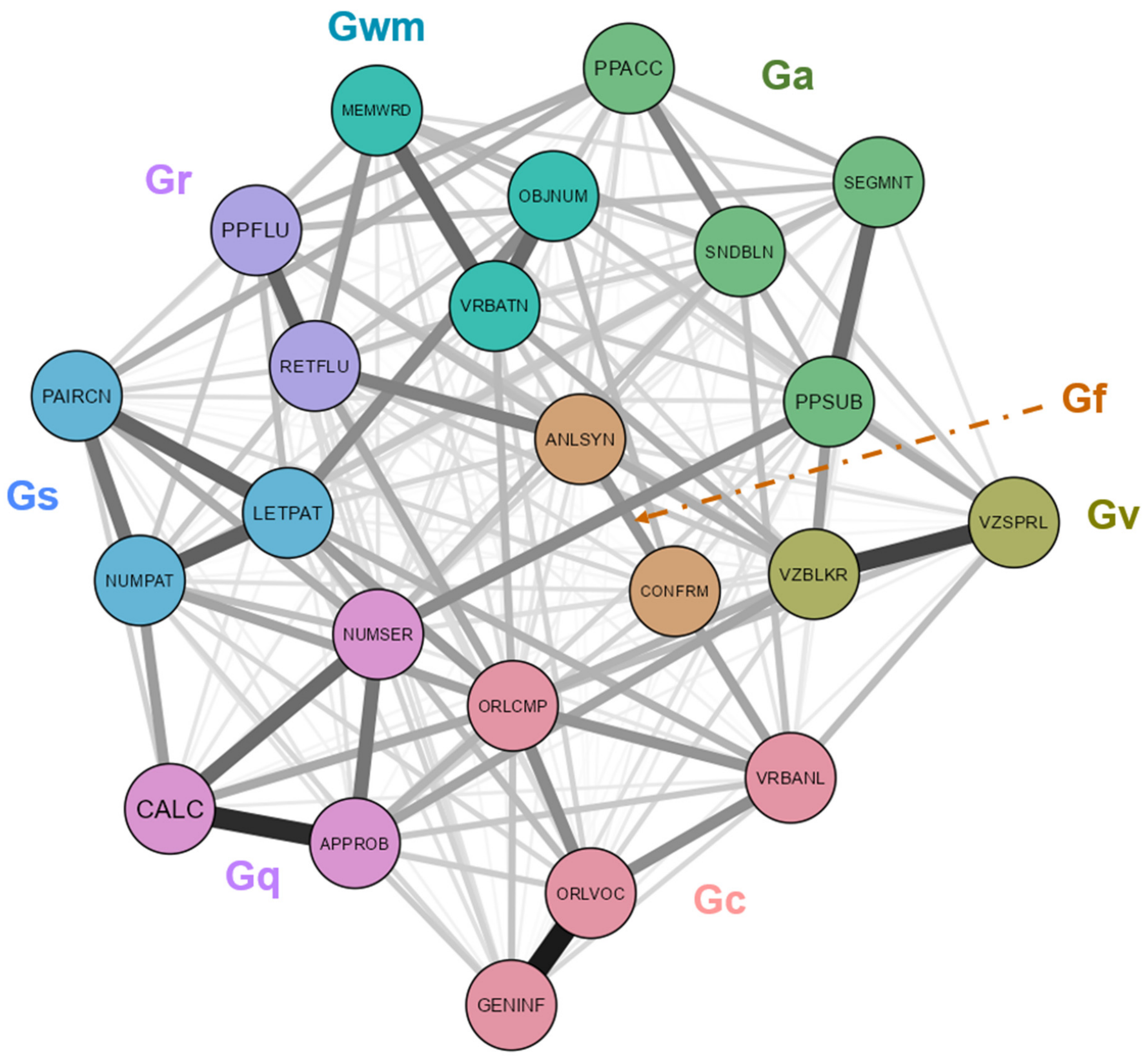

Figure S2. Weighted undirected network structure of 23 select WJ IV measures of eight broad CHC ability domains in the secondary sensitivity network model: JASP produced network model edited to include labels for CHC construct communities.

Section S3: Quantile Distribution of 20-measure Primary CHC Network Model

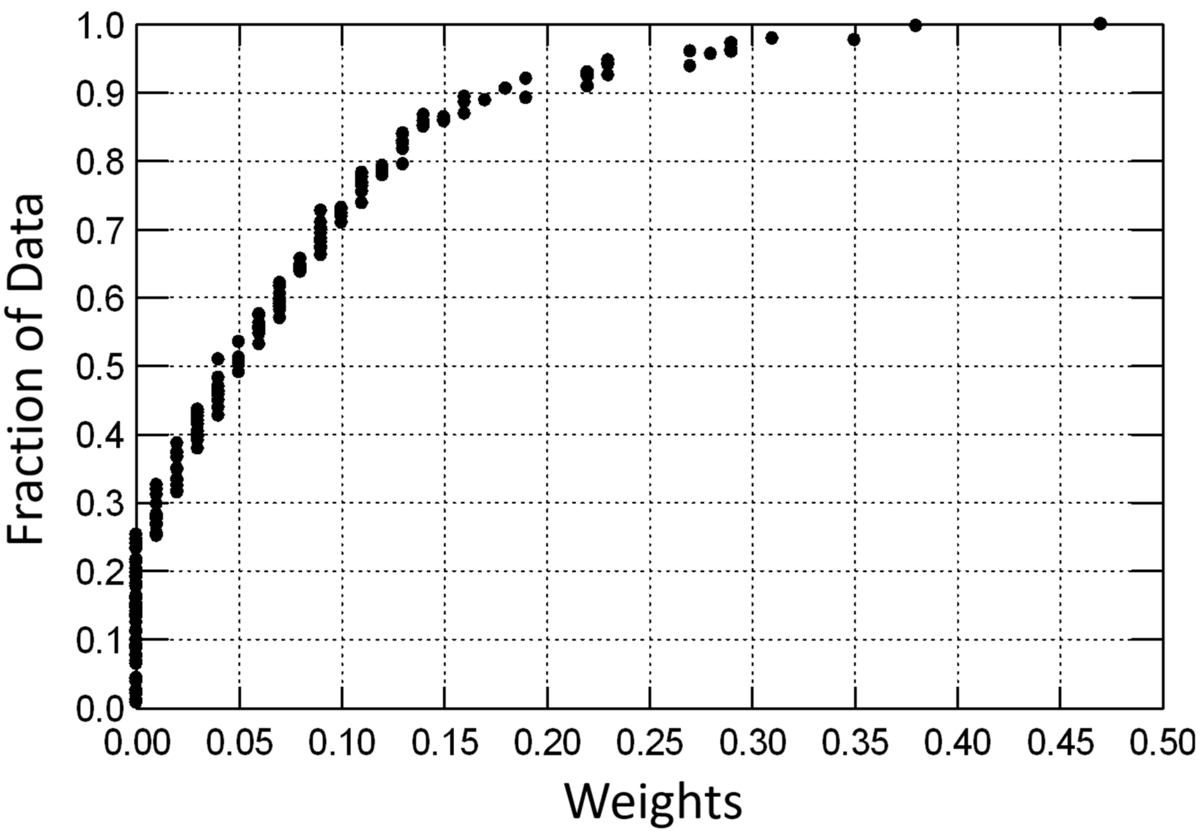

Figure S3. Quantile distribution of network edge weights from primary network model (Figure 1 and S1).

Section 4: Network Central Metric Stability Analysis

As per the recommendation of Epskamp et al. (2018a), the *case-dropping subset bootstrap* (n = 1000 samples) procedure, as operationalized in the *JASP* network module, was completed with the primary 20-measure CHC model to evaluate the stability of the network centrality metrics. These analyses indicate whether the relative ordering of centrality indices remain the same when the network is re-estimated with fewer cases. The stability metric is the average correlation of the re-estimated indices with the original complete sample indices, displayed as a function of percentage (%) of total sampled cases dropped. This term is called the *correlation stability coefficient* (*CS*-coefficient). The obtained sample means are displayed on each figure in with the X-axes indicating the % of the original sample size retained, along with the sample-based 95% confidence range (from the 2.5th quantile to the 97.5th quantile) around the mean values.

Figure S4 presents the *CS* case-dropping subset bootstrap of the network centrality values for the 20-measure primary CHC network model as a function of sample size subsets. Although there is no firm criterion, Epskamp et al. (2018) have suggested that for case-dropping subset bootstrap analyses, if the "correlation completely changes after dropping, say, 10% of the cases, then interpretations of centralities are prone to error" (p. 200). As seen in Figure S4, all CS values for the three centrality metrics remain high up to samples with 60% or more of cases dropped, suggesting stable network centrality indices.

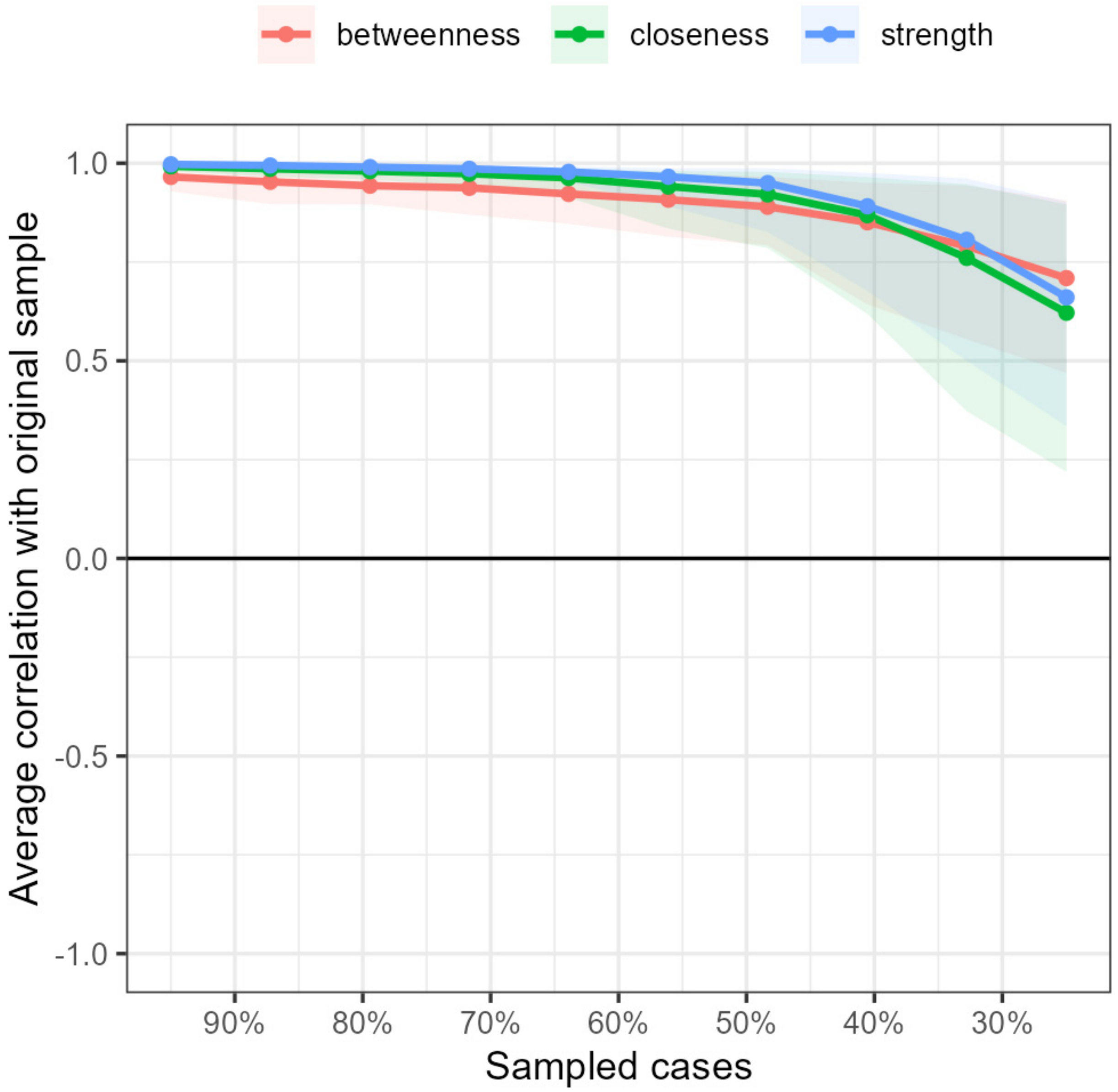

Figure S4. Case-dropping Boostrap (n = 1000) Correlation Stability (CS) Coefficients for 20-measure CHC Network Model Centrality Indices.

Section 5: 25-measure (Inclusive of Gl Measures) Sensitivity Analysis Results

Figures S5 and S6 present the psychometric network model and the MDS+MST figures for the second sensitivity analysis model that, in addition to including all measures from the 23-measure sensitivity model, also included the apriori excluded WJ IV Gl measures. In both Figures S5 and S6 the Visual-Auditory Learning and Story Recall measures fail to suggest a common dimension. They are clearly not close neighbors in the two visual network figures. These findings suggest that the robust seven CHC broad ability dimension model in the primary analysis is diminished by the inclusion of these two WJ IV Gl measures. These findings suggest either that the WJ IV Gl measures are not strong indicators of the broad Gl ability, or the definition and validity of the CHC broad Gl ability warrants additional scrutiny.

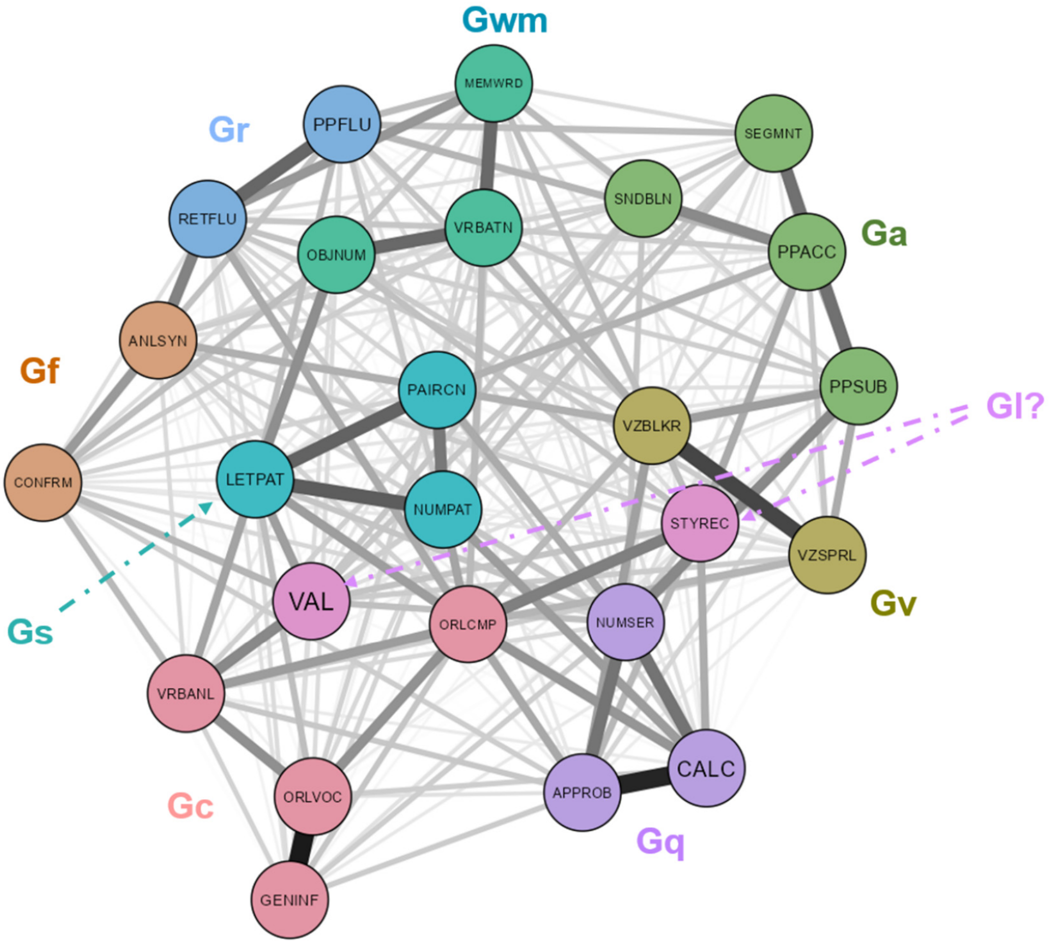

Figure S5. Weighted undirected network structure of 25 select WJ IV measures of nine broad CHC ability domains in secondary sensitivity network model.

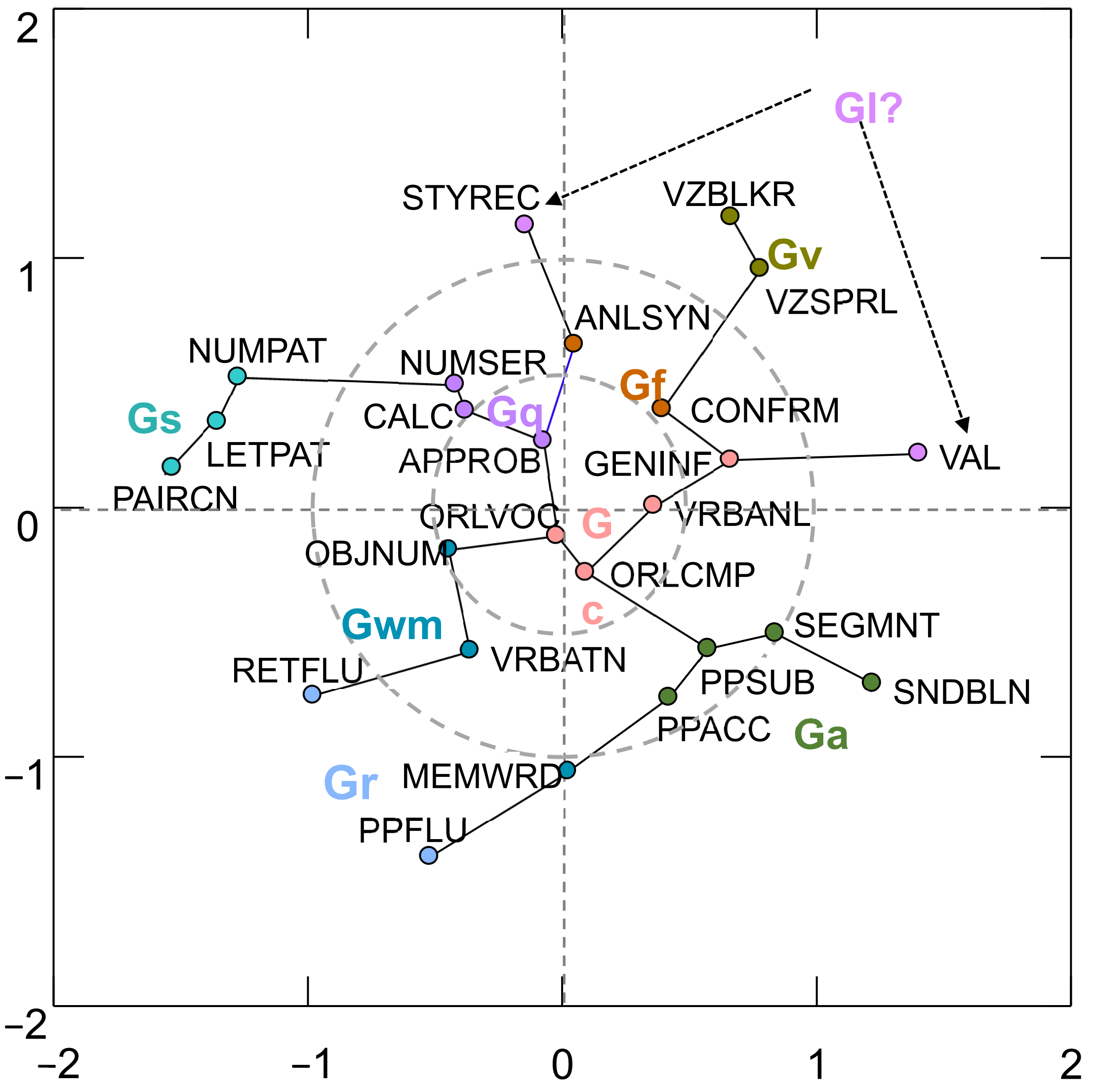

Figure S6. 2-D multidimensional scaling analysis (Guttman Radex) of 25-measure secondary sensitivity model connected by minimal spanning tree algorithm.

Section S6: Psychometric *g*-loadings for 20 Primary Measures

Table S1: Psychometric *g*-loadings for 20 primary measures.

			Psychometric *g*-loadings	
**WJ IV Measure**	**Abbrevia-tion**	**CHC domain**	***g*-PCA**	***g*-PAF**
Analysis-Synthesis	ANLSYN	Gf	0.65	0.62
Concept Formation	CONFRM	Gf	0.66	0.64

Verbal Analogies	VRBANL	Gc/Gf	0.76	0.75
General Information	GENINF	Gc	0.62	0.59
Oral Comprehension	ORLCMP	Gc	0.67	0.65
Oral Vocabulary	ORLVOC	Gc	0.76	0.74
Block Rotation	VZBLKR	Gv	0.53	0.50
Spatial Relations	VZSPRL	Gv	0.56	0.53
Phon. Proc.-Word Access	PPACC	Ga	0.65	0.63
Phon. Proc.-Substitution	PPSUB	Ga	0.64	0.61
Segmentation	SEGMNT	Ga	0.63	0.60
Sound Blending	SNDBLN	Ga	0.57	0.54
Phon. Proc.-Word Fluency	PPFLU	Gr	0.54	0.51
Retrieval Fluency	RETFLU	Gr	0.58	0.55
Object-Number Seq.	OBJNUM	Gwm	0.69	0.67
Memory for Words	MEMWRD	Gwm	0.63	0.60
Verbal Attention	VRBATN	Gwm	0.64	0.61
Letter-Pattern Matching	LETPAT	Gs	0.53	0.50
Number-Pattern Matching	NUMPAT	Gs	0.53	0.50
Pair Cancellation	PAIRCN	Gs	0.52	0.49
Number Series	NUMSER	Gq		
Applied Problems	APPROB	Gq		
Calculation	CALC	Gq		

Note. Correlations between measures respective *g*-loadings (PCA) and *g*-loadings (PAF) was .999 or approximately unity.

Section S7: Post-hoc 19-measure (Oral Comprehension excluded) Sensitivity Model AnalysisGiven the unexpected centrality of the Oral Comprehension measure, a post hoc sensitivity model analysis was completed with the Oral Comprehension measure excluded. The resulting 19-variable CHC network model is presented in Figure S7. The network centrality measure metrics for this model are presented in Table S2.

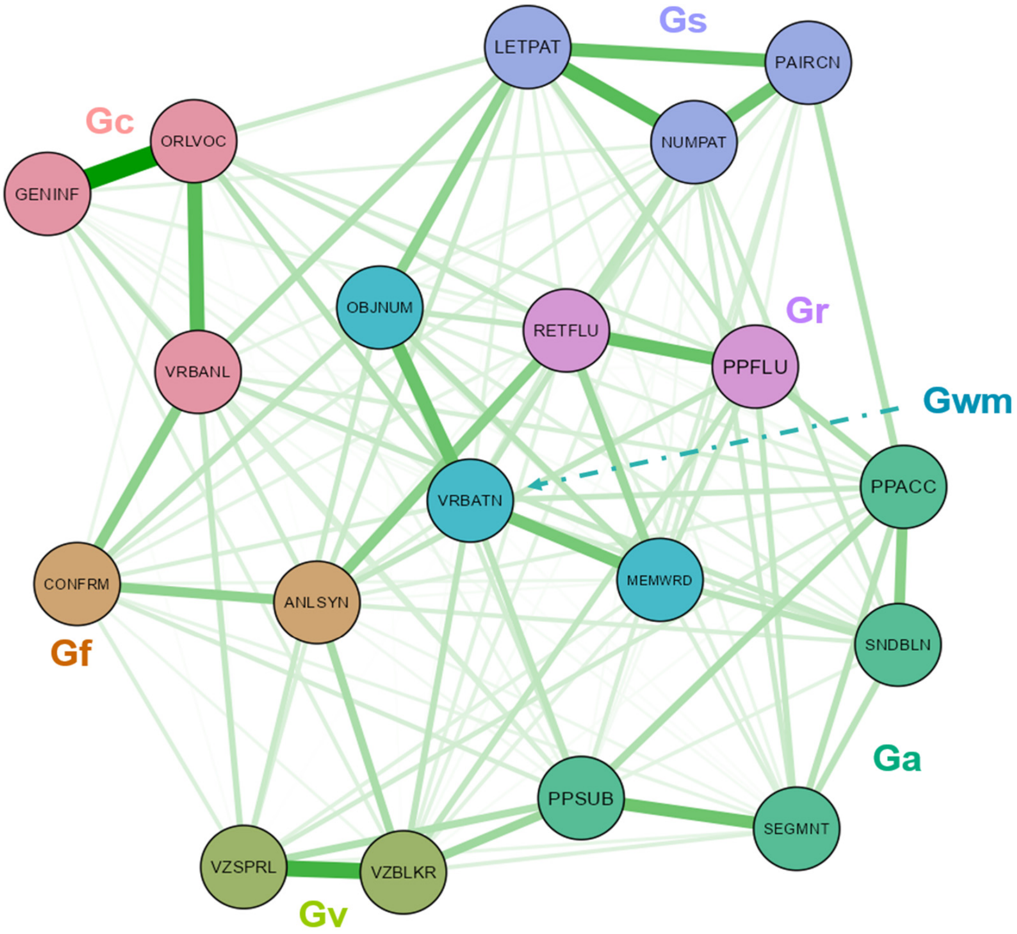

Figure S7. Weighted undirected network structure of 19 (Oral Comprehension excluded) select WJ IV measures of seven broad CHC ability domains in primary network model.

Table S2. 19-measure (Oral Comprehension excluded) primary sensitivity network model centrality measure metrics.

			Network relative centrality characteristic metrics		
			20 Variable Primary Model		
WJ IV Measure	Abbreviation	CHC domain	Betweeness	Closeness	Strength
Analysis-Synthesis	ANLSYN	Gf	0.30	0.86	0.82
Concept Formation	CONFRM	Gf	0.17	0.82	0.68
Verbal Analogies	VRBANL	Gc/Gf	**1.00**	**0.92**	0.88
General Information	GENINF	Gc	0.00	0.75	0.62

Oral Vocabulary	ORLVOC	Gc	**0.74**	0.83	0.88
Block Rotation	VZBLKR	Gv	0.30	0.81	0.83
Spatial Relations	VZSPRL	Gv	0.09	0.78	0.68
Phon. Proc. - Word Access	PPACC	Ga	0.22	0.85	0.79
Phon. Proc. - Substitution	PPSUB	Ga	0.48	0.83	**0.92**
Segmentation	SEGMNT	Ga	0.13	0.76	0.72
Sound Blending	SNDBLN	Ga	0.09	0.80	0.66
Phon. Proc. - Word Fluency	PPFLU	Gr	0.17	0.88	0.81
Retrieval Fluency	RETFLU	Gr	0.44	**0.94**	**0.92**
Object-Number Sequencing	OBJNUM	Gwm	0.35	0.91	0.76
Memory for Words	MEMWRD	Gwm	0.13	0.85	0.81
Verbal Attention	VRBATN	Gwm	**0.74**	**1.00**	**1.00**
Letter-Pattern Matching	LETPAT	Gs	**0.74**	**0.92**	**0.95**
Number-Pattern Matching	NUMPAT	Gs	0.17	0.85	0.85
Pair Cancellation	PAIRCN	Gs	0.09	0.80	0.70

Note. Bold font designates the top three (sometimes four) relative centrality values in each column.

Reference

(Bringmann et al. 2019) Bringmann, Laura F, Timon Elmer, Sacha Epskamp, Robert W. Krause, David Schoch, Marieke Wichers, Johanna T. W. Wigman, and Evelien Snippe. 2019. What do centrality measures measure in psychological networks? *Journal of Abnormal Psychology* 128: 892–903. https://doi.org/10.1037/abn0000446.

(Carroll 1993) Carroll, John B. 1993. *Human Cognitive Abilities: A Survey of Factor-Analytic Studies*. Cambridge: Cambridge University Press. https://doi.org/10.1017/cbo9780511571312.

(Comrey 1988) Comrey, Andrew L. 1988. Factor-analytic methods of scale development in personality and clinical psychology. *Journal of Consulting and Clinical Psychology* 56: 754-761.

(Epskamp et al. 2012) Epskamp, Sacha, Angélique O. J. Cramer, Lourens J. Waldorp, Verena D. Schmittmann, and Denny Borsboom. 2012. qgraph: Network Visualizations of Relationships in Psychometric Data. *Journal of Statistical Software* 48. doi.org/10.3758/s13428-017-0862-1.

(Epskamp et al. 2018a) Epskamp, Sacha, Denny Borsboom, and Eiko I. Fried. 2018a. Estimating psychological networks and their accuracy: A tutorial paper. *Behavior Research Methods* 50: 195–212. https://doi.org/10.3758/s13428-017-0862-1.

(McGrew et al. 2014) McGrew, Kevin S., Erica M. LaForte, and Fredrick A. Schrank. 2014. *Woodcock-Johnson IV Technical Manual*. Rolling Meadows: Riverside.

(McGrew and Flanagan 1998) McGrew, Kevin S., and Dawn P. Flanagan, D. P. 1998. *The intelligence test desk reference (ITDR): Gf-Gc cross-battery assessment*. Boston: Allyn & Bacon.

(Messick 1995) Messick, Samuel. 1995. Validity of psychological assessment: Validation of inferences from persons' responses and performances as scientific inquiry into score meaning. *American Psychologist* 50: 741-749.

(Neal and Neal 2021) Neal, Zachary P., and Jennifer W. Neal. 2021. Out of bounds? The boundary specification problem for centrality in psychological networks. *Psychological Methods*. Advance online publication. https://doi.org/10.1037/met0000426.

(Pedersen 2022a) Pedersen, Thomas L. 2022a. ggforce: Accelerating "ggplot2" [R package]. Available online: ggforce.data-imaginist.com (accessed on 20 August 2022)._

(Pedersen 2022b) Pedersen, Thomas L. 2022b. tidygraph: A Tidy API for Graph Manipulation [R Package]. Available online: https://tidygraph.data-imaginist.com (accessed on 20 August 2022)..

(Schneider 2016) Schneider, W. Joel. 2016. Strengths and weaknesses of the Woodcock-Johnson IV Tests of Cognitive Abilities: Best practice from a scientist-practitioner perspective. In *WJ IV Clinical Use and Interpretation*. Edited by Dawn P. Flanagan and Vincent C. Alfonso. Cambridge: Academic Press, pp. 191–210. https://do.org/10.1016/B978-0-12-802076-0.00007-4.

(Schneider and McGrew 2018) Schneider, W. Joel and Kevin S. McGrew. 2018. The Cattell-Horn-Carroll theory of cognitive abilities. In *Contemporary Intellectual Assessment: Theories, Tests and Issues*, 4th ed. Edited by Dawn P. Flanagan and Erin M. McDonough. New York: Guilford Press, pp. 73–130.

(Schrank et al. 2015) Schrank, Fredrick A., Kevin S. McGrew, and Nancy Mather. 2015. *Woodcock-Johnson IV Tests of Early Cognitive and Academic Development*. Rolling Meadows: Riverside.

(Wickham 2016) Wickham, Hadley 2016. *ggplot2: Elegant Graphics for Data Analysis*. Berlin/Heidelberg: Springer-Verlag._

(Wilke 2020) Wilke, Claus O. 2020. ggtext: Improved Text Rendering Support for ggplot2. Available online: CRAN.R-project.org/package=ggtext (accessed on 20 August 2022).
